# Public health implications of *Yersinia enterocolitica* investigation: an ecological modeling and molecular epidemiology study

**DOI:** 10.1186/s40249-023-01063-6

**Published:** 2023-04-21

**Authors:** Yuan Yue, Jinxin Zheng, Mei Sheng, Xiang Liu, Qiong Hao, Shunxian Zhang, Shuai Xu, Zhiguo Liu, Xuexin Hou, Huaiqi Jing, Yang Liu, Xuezhang Zhou, Zhenjun Li

**Affiliations:** 1 Key Laboratory of the Ministry of Education for the Conservation and Utilization of Special Biological Resources of Western China, Ningxia University, Yinchuan, People’s Republic of China; 2 State Key Laboratory for Infectious Disease Prevention and Control, National Institute for Communicable Disease Control and Prevention, Chinese Center for Disease Control and Prevention, Beijing, People’s Republic of China; 3 Ningxia Hui Autonomous Region Food Testing and Research Institute, Yinchuan, People’s Republic of China; 4 Department of Nephrology, Ruijin Hospital, Institute of Nephrology, Shanghai Jiao Tong University School of Medicine, Shanghai, People’s Republic of China; 5 School of Global Health, Chinese Center for Tropical Diseases Research-Shanghai Jiao Tong University School of Medicine, Shanghai, People’s Republic of China; 6 Ningxia Hui Autonomous Region Centre for Disease Control and Prevention, Yinchuan, People’s Republic of China; 7 Longhua Hospital, Shanghai University of Traditional Chinese Medicine, Shanghai, People’s Republic of China; 8 Department of Computer Science, Hong Kong Baptist University, Hong Kong, Special Administrative Region People’s Republic of China

**Keywords:** *Yersinia enterocolitica*, Ecological, Machine learning, Molecular epidemiology, Core genome multilocus sequence typing, Ningxia, China

## Abstract

**Background:**

*Yersinia enterocolitica* has been sporadically recovered from animals, foods, and human clinical samples in various regions of Ningxia, China. However, the ecological and molecular characteristics of *Y. enterocolitica*, as well as public health concerns about infection in the Ningxia Hui Autonomous Region, remain unclear. This study aims to analyze the ecological and molecular epidemiological characteristics of *Y. enterocolitis* in order to inform the public health intervention strategies for the contains of related diseases.

**Methods:**

A total of 270 samples were collected for isolation [animals (*n* = 208), food (*n* = 49), and patients (*n* = 13)], then suspect colonies were isolated and identified by the API20E biochemical identification system, serological tests, biotyping tests, and 16S rRNA-PCR. Then, we used an ecological epidemiological approach combined with machine learning algorithms (general linear model, random forest model, and eXtreme Gradient Boosting) to explore the associations between ecological factors and the pathogenicity of *Y. enterocolitis*. Furthermore, average nucleotide identity (ANI) estimation, single nucleotide polymorphism (SNP), and core gene multilocus sequence typing (cgMLST) were applied to characterize the molecular profile of isolates based on whole genome sequencing. The statistical test used single-factor analysis, Chi-square tests, *t*-tests/ANOVA-tests, Wilcoxon rank-sum tests, and Kruskal–Wallis tests.

**Results:**

A total of 270 isolates of *Yersinia* were identified from poultry and livestock (*n* = 191), food (*n* = 49), diarrhoea patients (*n* = 13), rats (*n* = 15), and hamsters (*n* = 2). The detection rates of samples from different hosts were statistically different (*χ*^2^ = 22.636, *P* < 0.001). According to the relatedness clustering results, 270 isolates were divided into 12 species, and *Y. enterocolitica* (*n* = 187) is a predominated species. Pathogenic isolates made up 52.4% (98/187), while non-pathogenic isolates made up 47.6% (89/187). Temperature and precipitation were strongly associated with the pathogenicity of the isolates (*P* < 0.001). The random forest (RF) prediction model showed the best performance. The prediction result shows a high risk of pathogenicity *Y. enterocolitica* was located in the northern, northwestern, and southern of the Ningxia Hui Autonomous Region. The *Y. enterocolitica* isolates were classified into 54 sequence types (STs) and 125 cgMLST types (CTs), with 4/O:3 being the dominant bioserotype in Ningxia. The dominant STs and dominant CTs of pathogenic isolates in Ningxia were ST429 and HC100_2571, respectively.

**Conclusions:**

The data indicated geographical variations in the distribution of STs and CTs of *Y. enterocolitica* isolates in Ningxia. Our work offered the first evidence that the pathogenicity of isolates was directly related to fluctuations in temperature and precipitation of the environment. CgMLST typing strategies showed that the isolates were transmitted to the population via pigs and food. Therefore, strengthening health surveillance on pig farms in high-risk areas and focusing on testing food of pig origin are optional strategies to prevent disease outbreaks.

**Supplementary Information:**

The online version contains supplementary material available at 10.1186/s40249-023-01063-6.

## Background

*Yersinia enterocolitica* is a common zoonotic pathogen that is widely found in soil, water, animals, and various foods [1, 2]. *Y. enterocolitica* is heterogeneous and consists of six biotypes: 1A, 1B, 2, 3, 4, and 5. Biotype 1B is regarded as highly pathogenic, and biotype 1A is regarded as being non-pathogenic in humans; other biotypes are considered to have low pathogenicity [3]. Enteric yersiniosis has been reported globally, with the most severe epidemic in Europe, where it was the fourth most common zoonotic disease in 2019 [4]. New Zealand has a high rate of yersiniosis notification, which is on the rise [5]. There were two outbreaks of *Y. enterocolitica* in China in the mid-1980s, resulting in over 500 infections [6]. Surveillance of the disease in China ceased for nearly 20 years, leading clinicians to rarely consider yersiniosis in the diagnosis of gastrointestinal diseases, and testers rarely being able to provide a basis for accurate diagnosis [7]. *Y. enterocolitica* was included in the national food contaminant surveillance network until 2016 [8]. The lack of mandatory government oversight in many countries may lead to an underestimation of the incidence of gastrointestinal disorders caused by *Y. enterocolitica* [9, 10]*.* Such cases reflect a general lack of attention to the disease at a domestic and international level, which can lead to untimely treatment and misdiagnosis, resulting in chronic and prolonged infection. As such, research on *Y. enterocolitica* is essential and urgent.

The emergence and re-emergence of zoonotic diseases have been recognized as being driven by variability at the human-animal-environment interface [11]. However, this comprehensive investigation is still lacking in almost all zoonotic systems, as we lack empirically validated models of ecological interactions between humans, animal reservoirs, and key environmental drivers [12]. In order to clarify the complexity of zoonotic spills, field monitoring and modelling methods that link human-animal contact with disease dynamics within animal reservoirs are essential [13]. The surveillance survey results of diseases caused by *Y. enterocolitica* in China through consecutive years have indicated that its hosts include livestock, poultry, and rodents [14]. Pigs are an essential source of *Y. enterocolitica* infections [15]. *Y. enterocolitica* is transmitted via the faecal-oral route as well as through contact with animals (including farm animals, domestic pets, and wild animals) and contaminated food [14, 16]. Previous studies have shown that the separation rate of domestic animals (pigs and dogs) was significantly and positively correlated with altitude, mean temperature, and precipitation, significantly and negatively correlated with altitude and mean temperature, and significantly and positively correlated with rainfall [14]. However, the integrated picture of host-environment interactions and the resulting spread and spillover of *Y. enterocolitica* is far from clear. Yersiniosis is an example of a health threat that affects humans, animals, and our shared environment, and controlling outbreaks of yersiniosis highlights the importance of a sustainable One Health approach. A crucial component of the One Health approach is identifying and monitoring the prevalence of zoonotic diseases in an area. However, information about the epidemiology of *Y. enterocolitica* in Ningxia is scarce. In this area, *Y. enterocolitica* has been sporadically recovered from animals, foods, and human clinical samples.

The primary objective of this study was to explore the ecological factors and molecular characteristics that influence the spread and pathogenicity of *Y. enterocolitica*. An exogenous association between ecological factors and the pathogenicity of isolates in Ningxia was demonstrated using a predictive modelling approach. The distribution and molecular characteristics within *Y. enterocolitica* populations in the region were explored through correlated genomic analyses. The public health implications of the host–human–environment relationship were explored in terms of the endogenous and exogenous causes of *Yersinia* transmission and provided a reference for the tracking and control of related zoonotic diseases. Health threats at the human–animal–environment interface are best addressed through an efficient and sustainable One Health approach.

## Methods

### Data sources

The 270 isolates in this experiment were obtained from five prefectures in Ningxia Hui Autonomous Region (Yinchuan, Shizuishan, Wuzhong, Guyuan, and Zhongwei) from 2007 to 2019. The isolates were obtained from animals, food, and patients. Animal hosts included pigs, sheep, rats, cattle, chickens, and hamsters. Pharyngeal swabs, anal swabs, intestinal contents, faeces and food samples were collected. Patient samples were obtained from faeces of diarrhoea patients collected at monitoring sites. Collection of pharyngeal swabs, anal swabs, and intestinal contents from healthy sheep and fattening pigs in slaughters. Pharyngeal swabs, anal swabs, and faeces collected from animals on farms. Intestinal contents of rats and hamsters from hunted rats and hamsters. Foods were purchased at butchers, retail markets and supermarkets in the region. All samples were transported to the laboratory in separate sterile containers using a cooler. Isolates enrichment was performed in phosphate-buffered saline with sorbitol and bile salts (PSB) at 4 ℃ for 21 days.

Environmental and climatic data obtained from open-access data sources, including elevation, normalized difference vegetation index (NDVI), monthly mean temperature, and rainfall (Additional file 8: Table S1). The NDVI and elevation data were aligned over a grid with a spatial resolution of 5 × 5 km. Data from the survey samples were extracted from the corresponding geo-referenced locations. The data processing procedures described above were using software R 4.6.0 (Lucent Technologies, Jasmine Mountain, USA) with the ‘raster’ package.

### Collection and identification of isolates

*Yersinia* was inoculated onto selective agar (CIN Agar; Oxoid, Basingstoke, UK/HKM, Guangzhou, PRC). A typical bulls-eye appearance (deep red centers surrounded by outer transparent zones) on CIN-selective agar plates was inoculated onto Kligler iron and urea media. We performed the identification of the isolates using the API20E biochemical identification system. The 16SrRNA fragment was amplified to confirm the species. The isolates were further distinguished by serotyping (*Y. enterocolitica* antisera set from the Institute of Chinese Biomedicine) [17] and biotyping (Bile Aesculin Agar; Oxoid, Basingstoke, UK. Brain Heart Infusion Agar; Oxoid, Basingstoke, UK. Tween 80; Amresco, USA. CaCl_2_; Sinopharm Chemical Reagent Co., Ltd, PRC. Biochemical Reaction Tablets; Rosco, Denmark) [19]. The biotype was determined by biochemical experiments on lipase activity, salicin, esculin hydrolysis, xylose, trehalose, indole production, ornithine decarboxylase, Voges-Proskauer test, pyrazinamidase activity, sorbose, inositol, and nitrate reduction [18, 19].

### Whole-genome sequencing and assembly

Genomic DNA was extracted using a Wizard® Genomic DNA Purification Kit (Promega, United States) according to the manufacturer’s protocol. DNA concentration, quality, and integrity were determined using a 5400 Fragment Analyzer System (Agilent, United States) and a NanoDrop 2000 Spectrophotometer (Thermo, United States). All 270 isolates were sequenced using Illumina Nova technology. Raw reads were quality filtered and assembled. The conditions for filtering were as follows: (1) reads containing more than 40% low-quality bases (mass value ≤ 20) were removed; (2) reads with N beyond a certain proportion (the default was 10%) were removed; (3) reads that overlapped with the adapter sequence by more than 15 bp with fewer than three mismatches between them were removed. Assemblies were performed using SOAP denovo (http://soapdenovo2.sourceforge.net/) [20], SPAdes (http://bioinf.spbau.ru/spades), and Abyss (https://www.bcgsc.ca/resources/software/abyss) software. Assembly results were integrated using CISA software (http://sb.nhri.org.tw/CISA/en/CISA). The preliminary assembly results were complemented with gap-closing software to filter reads with low sequencing depths (less than 0.35 of the average depth) to remove homolane contamination, resulting in the final assembly results. The reference genomes of 26 species of the *Yersinia* genus were shown in Additional file 8: Table S2. The ANI was estimated for each pair of isolates using the 270 *Yersinia* genomes, the 26 reference genomes (see above), and fastANI v1.33 (https://github.com/ParBLiSS/FastANI) [21]. The reference genes for the *Yersinia* were the same as those listed in the previous section.

### Phylogenetic and pan-genome analyses

*Y. enterocolitica* 8081 (GCA_000009345.1) was used as the reference sequence, and the whole-genome sequences of 270 isolates were compared with the reference sequence using MUMmer 4.0 software (http://mummer.sourceforge.net/) [22]. All SNP loci of each strain were obtained. The following protocol was used: Integrate the position data of all SNP loci in the reference genome, filter SNPs from duplicated regions, and remove low-quality SNPs for subsequent analysis [23, 24]. Maximum likelihood (ML) trees were constructed using iQ-TREE (http://www.iqtree.org/) with 1000 bootstrap replicates. In addition, the whole genome sequences of 88 publicly available *Y. enterocolitica* isolates were downloaded from the NCBI database (https://www.ncbi.nlm.nih.gov/). These isolates were isolated from 11 countries: the United Kingdom (*n* = 36), New Zealand (*n* = 19), France (*n* = 10), Germany (*n* = 8), the United States (*n* = 3), Italy (*n* = 4), Ireland (*n* = 2), China (*n* = 2), Spain (*n* = 1), Greece (*n* = 1), and Australia (*n* = 1); and 1 unspecified as shown in Additional file 8: Table S3. An ML tree of *Y. enterocolitica* (this study and NCBI database) was constructed as the reference genome *Y. enterocolitica* 8081 (GCA_000009345.1) using roary v3.13.0 software (https://github.com/sanger-pathogens/Roary). The trees were then visualized and annotated in iTOL (https://itol.embl.de/) (using their associated scripts that were obtained after processing the previous series) [24].

### Virulence profiles

The assembled genomes of the isolates in this study were inserted into the Virulence Factor Database (VFDB; MOH Key Laboratory of Systems Biology of Pathogen, Institute of Pathogen Biology, Beijing, http://www.mgc.ac.cn) with default parameter selection.

### ST and core genome MLST determination

Sequence types (STs) and cgMLST (CTs) of *Y. enterocolitica* were assigned using EnteroBase (http://enterobase.warwick.ac.uk/species/index/yersinia) [25]. The McNally 7 Gene scheme contained *aar*F, *dfp*, *gal*R, *gln*S, *hem*A, *rfa*E, and *spe*A [26]. CgMLST scheme, cgMLST V1 + HeirCC V1 (Hierarchical cgMLST clustering) was based on the profiles of 1553 coding loci. The minimum spanning tree (MST) and neighbor-joining (NJ) trees were constructed using isolates from the Ningxia region and those publicly available in the NCBI database with GrapeTree v2.1 (https://github.com/achtman-lab/GrapeTree).

### Statistical analysis

Single-factor analysis was conducted to compare the differences of ecological environment factors between the host, biotype, serotype, and pathogenicity of *Yersinia*. Chi-square tests were used for categorical variables, while *t*-tests or ANOVA-tests were applied for normal distribution of continuous variables. If there exists non-normal distribution, Wilcoxon rank-sum tests and Kruskal–Wallis tests were used. The significance level was set to 0.05. The study also using the ecological environment factors to predict the risk of pathogenicity of *Yersinia.* We have split our data into training dataset (80%) and testing dataset (20%), and utilized general linear model (GLM), random forest model (RF), and XGBOOST (eXtreme Gradient Boosting) models to evaluate methodological improvements (Additional file 8: Table S4). The area under the receiver operating characteristic (ROC) curve was used to evaluate the model performance. The model was simulated by ‘caret’ package, and AUC (area under the curves) was performed by ‘PRROC’ package with statistical software R 4.6.0.

## Results

### Epidemic profile of *Yersinia* during 2007–2019

A total of 9031 samples were monitored from 2007 to 2019, with the detection rate of *Yersinia* ranging from 0.9% to 7.6% (Table 1). The highest detection rate was in 2014 (7.6%), eightfold higher than in 2013 (0.5%). The difference in positivity rates between years was statistically significant (*χ*^2^ = 40.282, *P* < 0.001). No significant upward or downward trend in detection rates between years. Samples from different hosts were detected from 0.3% to 5.9%, with the highest detection rate of 5.9% for samples from poultry and livestock sources (Table 2). The detection rates of samples from different hosts were statistically different (*χ*^2^ = 22.636,* P* < 0.001).

**Table 1:** Detection rates of *Yersinia*, 2007–2019 in Ningxia Hui Autonomous Region, China

Year	Sample size	Positive samples	Positive rate (%)
2007	539	18	3.3
2008	118	2	1.7
2009	816	39	4.8
2010	660	7	1.1
2011	959	37	3.9
2012	1327	28	2.1
2013	217	2	0.9
2014	384	29	7.6
2015	578	14	2.4
2016	630	8	1.3
2017	852	20	2.4
2018	899	36	4.0
2019	1052	30	2.9

**Table 2:** Detection rates of *Yersinia* in samples from different hosts, 2007–2019 in Ningxia Hui Autonomous Region, China

Host	Sample size	Positive samples	Positive rate (%)	Sample source
Livestock and poultry	3219	191	5.9	Feces, pharyngeal swabs, anal swabs, and intestinal contents
Food	511	49	3.7	Smear swabs
Diarrhoea patients	3824	13	0.3	Feces
Rat	1190	15	1.3	Intestinal contents
Hamster	287	2	0.7	Intestinal contents

**Fig. 1:**
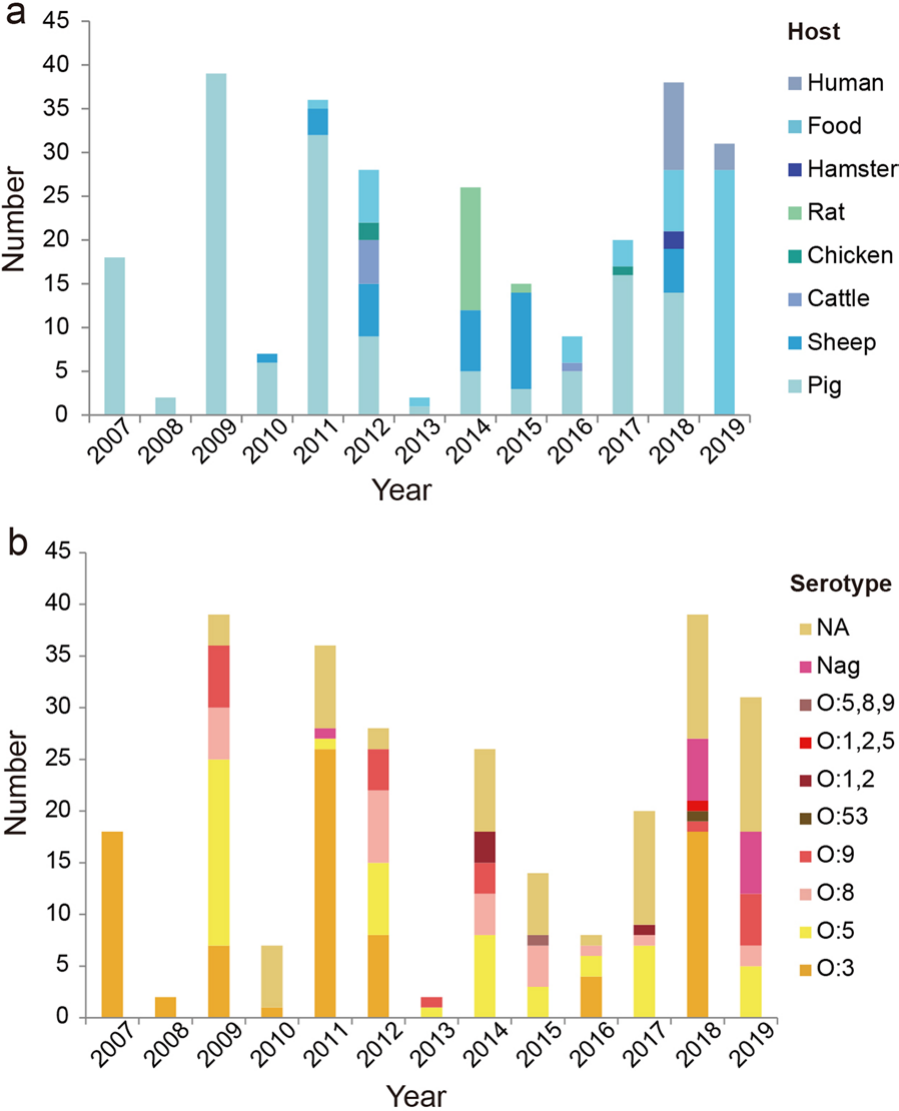
Number of *Yersinia* spp. isolates isolated from the Ningxia Hui Autonomous Region between 2007 and 2019. **a** Host distribution of isolates. **b** Serotype distribution of isolates. NA, not applicable; Nag, nonagglutinative

The isolates were obtained from five prefectures in Ningxia Hui Autonomous Region [Yinchuan (*n* = 111), Shizuishan (*n* = 7), Wuzhong (*n* = 1), Guyuan (*n* = 3), and Zhongwei (*n* = 148)], from 2007 to 2019 (Fig. 1 and Additional file 8: Table S5). In total, 208 (77.0%) were of animal origin, 49 (18.2%) were of food origin, and 13 (4.8%) were of patient origin. Animal hosts included pig (150/208, 72.1%), sheep (32/208, 15.4%), rat (15/208, 7.2%), cattle (6/208, 2.9%), chicken (3/208, 1.4%), and hamster (2/208, 1.0%). The source of animal samples was mainly feces (*n* = 102), pharyngeal swabs (*n* = 39), anal swabs (*n* = 7), and intestinal contents (*n* = 59) of animals. Food was derived from meat products, comprising beef (*n* = 25), pork (*n* = 10), chicken (*n* = 9), lamb (*n* = 3), and fish (*n* = 2). Food (*n* = 49) comprised fresh meat (*n* = 18) and frozen meat (*n* = 31). Human samples were of fecal origin, with the majority (*n* = 9) coming from children (Fig. 1 and Additional file 8: Table S5). Using traditional phenotypic methods, 187/270 (69.3%) isolates were serotyped, with the most common serotypes being O:3 (*n* = 84), O:5 (*n* = 52), O:8 (*n* = 24), and O:9 (*n* = 20). In total, 83 isolates were reported as O: unidentifiable because the O-antigen reacted with more than one antiserum or with none of the antisera (Fig. 1).

### Distribution of *Y. enterocolitica* by biotype, host, and serotype

Of the 187 *Y. enterocolitica* isolates, 81.3% (*n* = 152) were of animal origin, the food source was 12.3% (*n* = 23) and the patient source was 6.4% (*n* = 12). Isolates of animal origin included 42.8% biotype 1A (*n* = 65), 50.0% biotype 4 (*n* = 76), 2.6% biotype 3 (*n* = 4) and 4.6% biotype 5 (*n* = 7). Of these, all isolates of biotype 5 were from sheep. In total, biotype 4 of isolates (*n* = 84) were from pig hosts. The dominant serotype of the isolates of animal origin was O:3. Food-derived isolates were 87.0% biotype 1A (*n* = 20) and 13.04% biotype 2 (*n* = 3). Patient-origin isolates included 33.3% biotype 1A (*n* = 4) and 66.7% biotype 4 (*n* = 8). There was no biotype 1B (Table 3 and Additional file 8: Table S6).

**Table 3:** Serotype and biotype distribution of *Y.enterocolitica* isolates

		Animal (*n* = 152)					Food (*n* = 23)	Human (*n* = 12)
		Pig (*n* = 121)	Sheep (*n* = 17)	Cattle (*n* = 6)	Rat (*n* = 5)	Chicken (*n* = 3)		
Biotype	1A	41/33.9%	10/58.8%	6/100%	5/100%	3/100%	20/87.0%	4/33.3%
	2	0	0	0	0	0	3/13.0%	0
	3	4/3.3%	0	0	0	0	0	0
	4	76/62.8%	0	0	0	0	0	8/66.7%
	5	0	7/41.2%	0	0	0	0	0
Serotype	O:3	78/64.5%	0	0	0	0	0	5/41.7%
	O:5	30/24.8%	2/11.8%	6/100%	5/100%	2/66.7%	6/26.1%	0
	O:8	5/4.1%	12/70.6%	0	0	1/33.3%	5/21.7%	1/8.3%
	O:9	8/6.6%	2/11.8%	0	0	0	8/34.8%	1/8.3%
	O:53	0	0	0	0	0	1/4.4%	0
	O:1,2,5	0	0	0	0	0	0	0
	O:5,8,9	0	1/5.9%	0	0	0	0	1/8.3%
	Nag	0	0	0	0	0	3/13.0%	4/33.3%

The data indicate the number of isolates and the percentage of them.* Nag* nonagglutinative

### Phylogenetic analysis

**Fig. 2:**
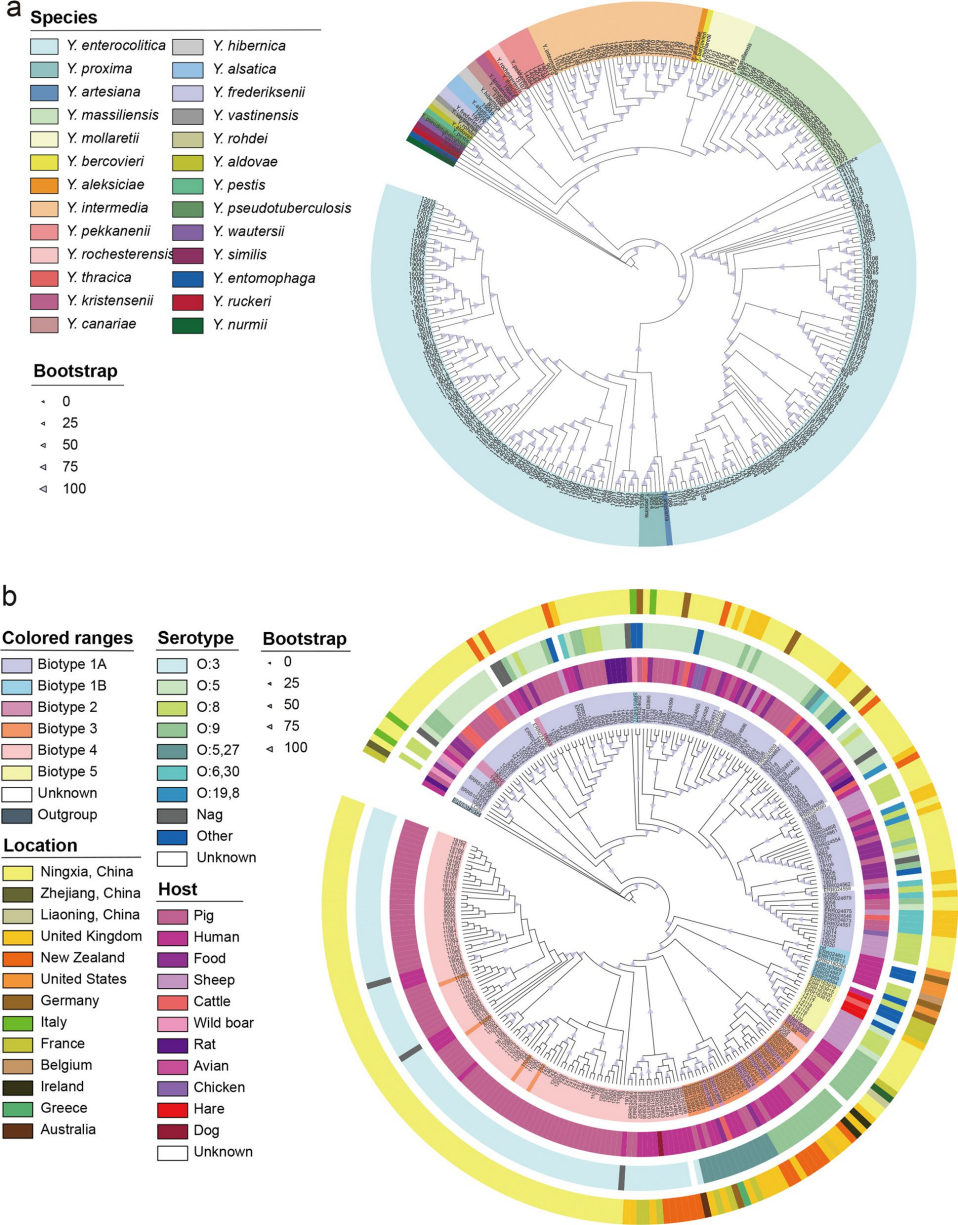
SNP-based maximum likelihood (ML) trees of *Yersinia* spp. isolates. **a** The SNP-based ML tree was built from a recombination-filtered alignment of the whole genome SNP (wgSNP) present in 270 isolates. The ML tree was built using the GTR + F + ASC + G4 model, with 1000 bootstraps based on 1,563,073 SNPs, with *Y. enterocolitica* 8081 (GCA_000009345.1) was used as the reference sequence. **b** The cgSNP-based ML tree was built from 187 *Y. enterocolitica* isolates of this study and 88 public data. *Y. enterocolitica* 8081 (GCA_000009345.1) was used as the reference sequence. *Y. pseudotuberculosis* (GCA_900637475.1) was used as outgroups

Total of 270 *Yersinia* genomes were evaluated according to the standard 95–96% ANI [27]. Twelve species were delineated using a 95% ANI cut-off value: *Y. enterocolitica* (187/270, 69.3%), *Y. intermedia* (30/270, 11.1%), *Y. massiliensis* (30/270, 11.1%), *Y. mollaretii* (7/270, 2.6%), *Y. pekkanenii* (5/270, 1.9%), *Y. proxima* (4/270, 1.5%), *Y. alsatica* (2/270, 0.7%), *Y. frederiksenii* (1/270, 0.4%), *Y. kristensenii* (1/270, 0.4%), *Y. hibernica* (1/270, 0.4%), *Y. canariae* (1/270, 0.4%), *and Y. rochesterensis* (1/270, 0.4%) (Fig. 2). The clustering analyses was consistent with the results of the ANI analysis and identical separation into 12 distinct species as determined by BAPS (Bayesian analysis of population structure) (Fig. 2).

The relatedness clustering presented by the *Y. enterocolitica* ML tree showed a direct relationship with the biotype. Biotypes 1A, 1B, and 5 isolates formed discrete clusters, whereas biotypes 2, 3, and 4 isolates consisted of closely related but distinct lineages, confirmed by BAPS clustering. Pathogenic isolates from Ningxia were genetically more distant from the reference genome than isolates from other countries and regions (no biotype 1B isolates in Ningxia). Isolate LC20 from Zhejiang had the longest genetic distance with the reference genome compared to the other isolates in China. Biotype 1A isolates exhibited no geographical differences and a broader range of host species and serotypes. The hosts of biotypes 4 and 5 isolates in Ningxia were pigs and sheep, respectively, whereas the hosts in the other regions were humans and hares. Biotype 3 isolates were O:3 in Ningxia, compared to O:5,27 and O:6,30 in the other countries (Fig. 2).

### Associations between ecological factors and pathogenicity

**Fig. 3:**
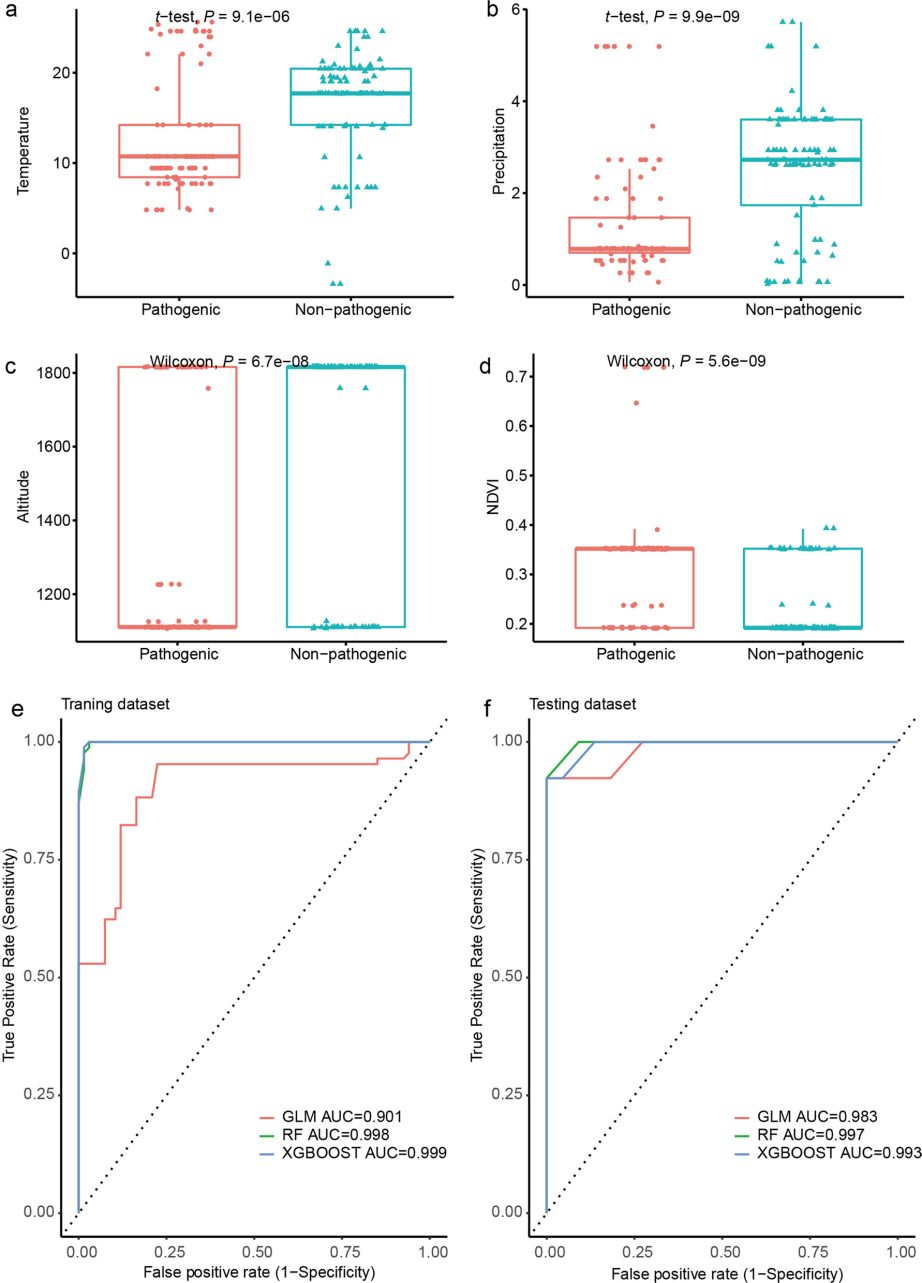
Differences in pathogenicity of *Yersinia enterocolitica* isolates and ecological factors and the development of predictive models. **a** Temperature. **b** Precipitation. **c** Altitude. **d** NDVI. **e** Training dataset. **f** Testing dataset

Temperature, precipitation, altitude, and NDVI were highly statistically significant to pathogenicity (*P* < 0.001) (Fig. 3a–d). The ambient temperature of the collection locations for pathogenic isolates was lower than that of the non-pathogenic isolates (12.70 ± 6.39 ℃ vs. 16.85 ± 6.05 ℃); the precipitation of the collection locations for the pathogenic isolates was lower than that of the non-pathogenic isolates (1.34 ± 1.29 mm vs. 2.54 ± 1.42 mm). The median elevation of the collection locations for pathogenic and non-pathogenic isolates was 1111.00 m and 1816.00 m, respectively; the median NDVI was 0.35 and 0.19, respectively. Also, except for the host of the isolates in precipitation factors, the ecological factors and pathogenicity were statistically highly significant with biotype, serotype of the isolates, and host of the isolates (Fig. 3, Additional file 1: Fig. S1, Additional file 2: Fig. S2, Additional file 3: Fig. S3).

Then, we used three models to predict the pathogenic of *Y. enterocolitica* with ecological environment factors. The AUC of the training and test sets of all three models exceeded 0.9, particularly the AUC of RF, and XGBOOST, indicating good evaluation performance (Fig. 3e, f). The contributions of ecological factors to the risk of *Y. enterocolitica* pathogenicity were shown in Additional file 4: Fig. S4, which indicated the prediction of *Y. enterocolitica* pathogenicity was the combination of all ecological factors. Finally, we used those performed models to evaluate the risk of *Y. enterocolitica* pathogenicity among our study regions (Additional file 5: Fig. S5). The results indicated that the highest risk area was concentrated in Yinchuan City and Shizuishan City in northern Ningxia, Zhongning City, and Zhongwei County in central Ningxia, and the southeastern part of Guyuan City and Haiyuan County in southern Ningxia. The predicted risk also varied between sources, with animals and humans suggesting a wider range of risk areas.

### Virulence profiles

**Fig. 4:**
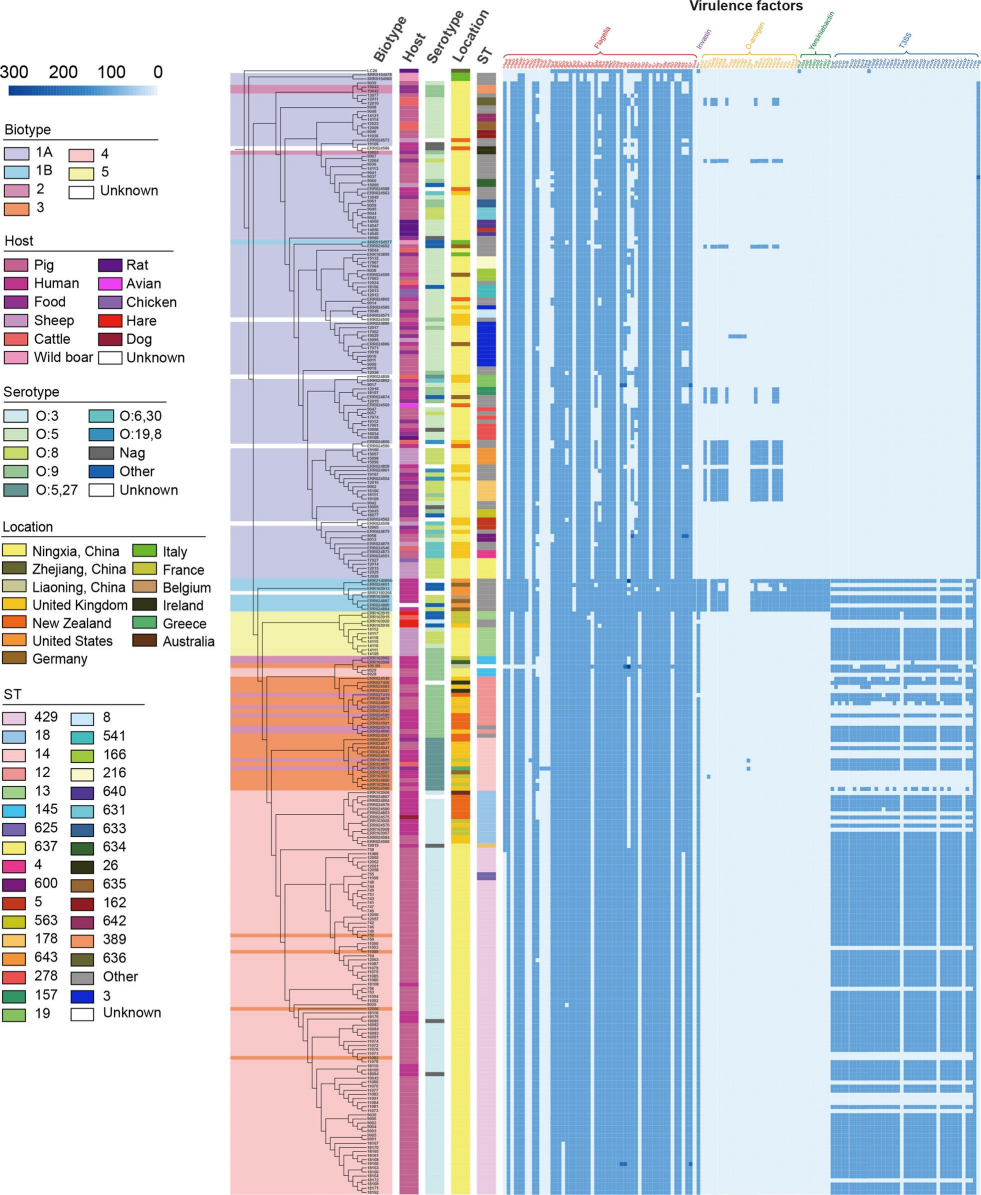
Distribution of virulence genes in *Yersinia enterocolitica* isolates. The heat map on the right depicted the host, serotype, STs, location, and virulence genes of the isolates

*Y. enterocolitica* isolates were annotated with 130 virulence genes in 5 categories: flagella, invasion, O-antigen, Yersiniabactin, and T3SS (Fig. 4). There were 53 flagella genes, most of which were *che*, *flh*, *flg* and *fli* genes. The O-antigen was represented by 27 genes, mostly *mrk* and *wbc* genes. The Yersiniabactin was made up of 9 genes, the majority of which were *ybt* gene. T3SS included 41 genes. The major ones were *ysc*, *yop*, *icr*, and *syc* genes. The distribution of virulence factors was closely related to the biotype. Most biotype 1A isolates lacked the virulence factors of invasion, O-antigen, Yersiniabactin, and T3SS. In contrast to pathogenic isolates of other biotypes, biotype 1B isolates were presented in Yersiniabactin.

### ST and cgMLST typing

**Fig. 5:**
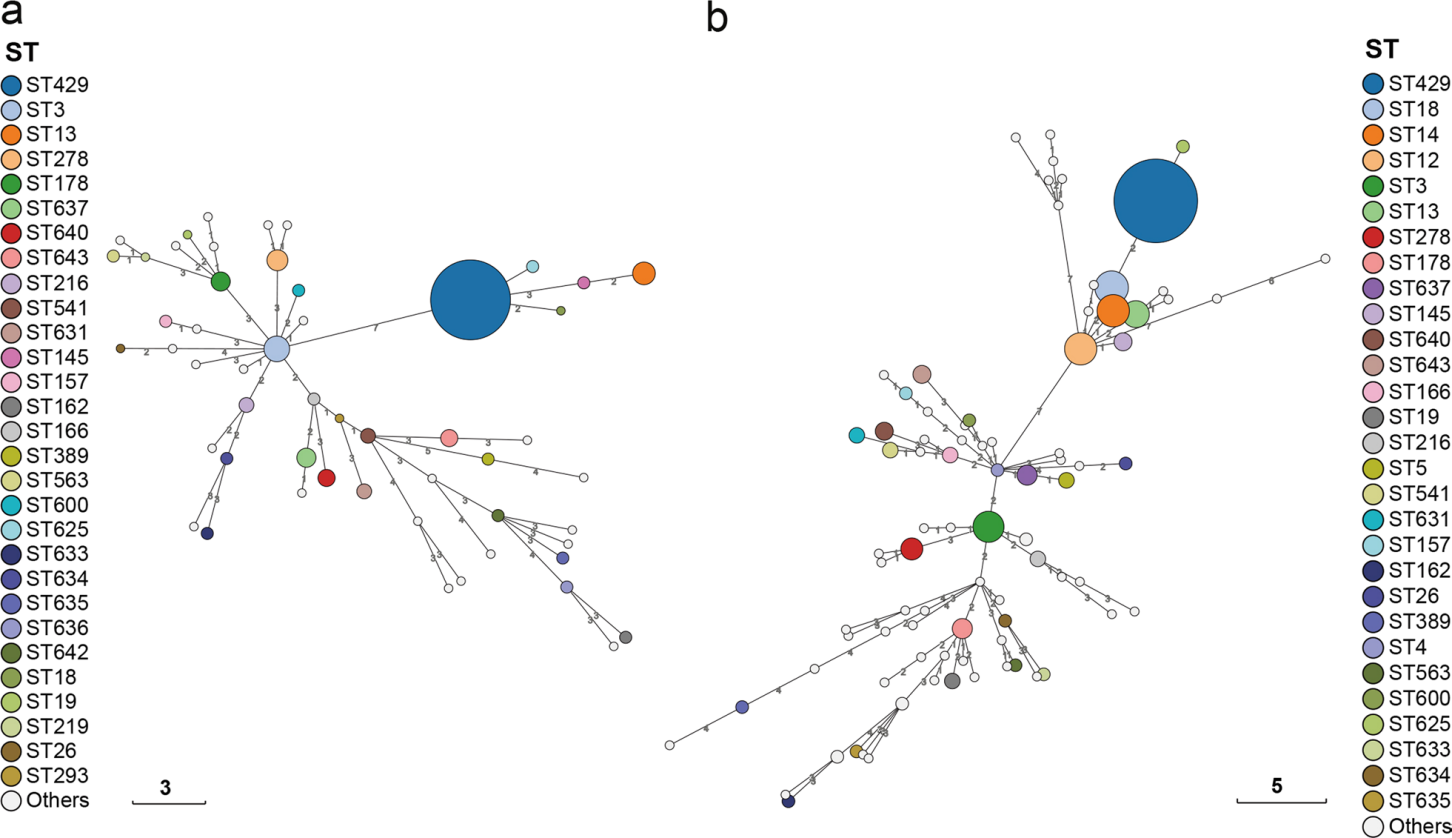
The minimum spanning trees (MST) of *Yersinia enterocolitica* isolates. The circle size was proportional to the number of isolates. Links between circles were represented according to the number of allelic differences between STs. **a** Isolates from this study. **b** Isolates from this study and the public database

In 187 *Y. enterocolitica* isolates, 54 STs were detected using multilocus sequence typing. ST429 was the most common, accounting for 42.3% of all STs (79/187) (Fig. 5 and Additional file 8: Table S6). ST429 isolates included biotype 4 (*n* = 79) and biotype 3 (*n* = 2), and they were closely related to serotype O:3. ST3 (*n* = 9), ST278 (*n* = 6), ST178 (*n* = 5), ST637 (*n* = 5), ST640 (*n* = 4), ST643 (*n* = 4), and ST216 (*n* = 3) were biotype 1A isolates with serotypes O:5, O:8, O:9. ST13 isolates (*n* = 7) were biotype 5, with serotypes O:5, O:8, and O:9. ST3, ST12, ST14, ST18, and ST13 were the principal STs of foreign isolates, which differed from those in the Ningxia region (Additional file 8: Tables S3, S6, and Fig. 5). The biotypes and serotypes corresponding to ST12 and ST4 were bioserotypes 1A/O:6,30 and 2,3,4/O:9 (Additional file 6: Fig. S6).

**Fig. 6:**
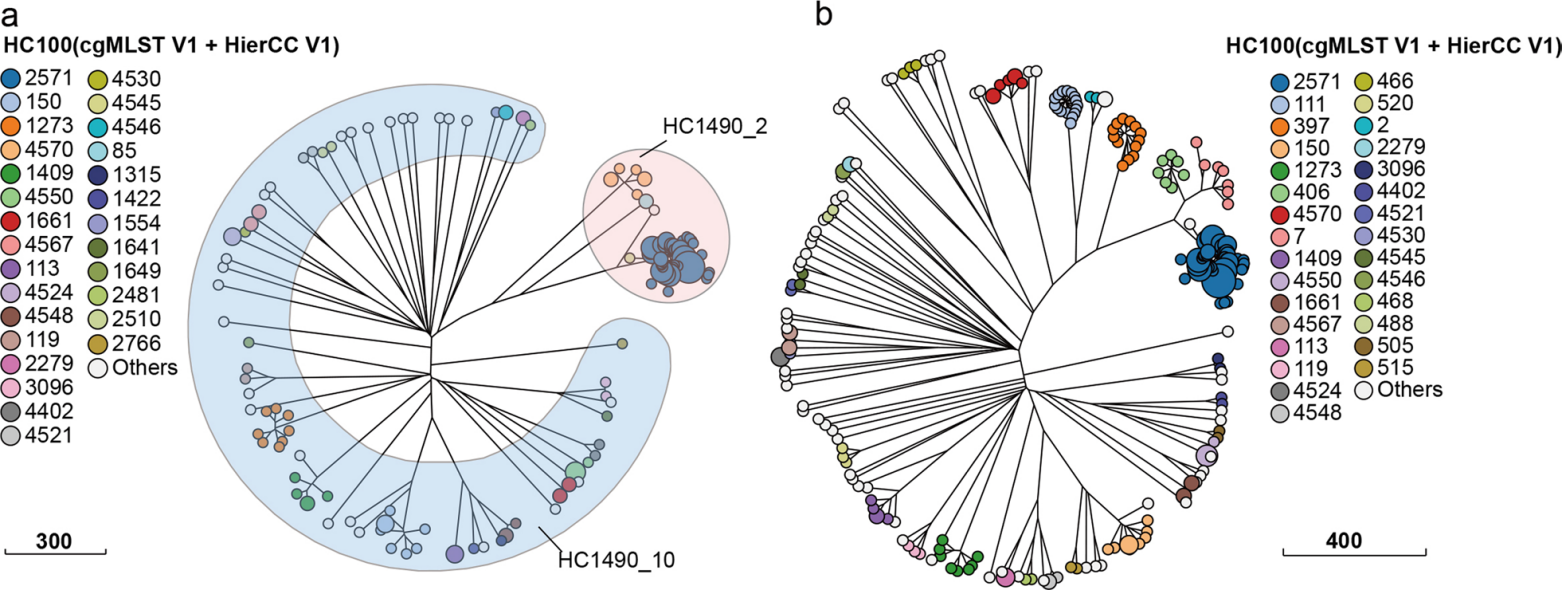
The neighbor-joining (NJ) tree of *Yersinia enterocolitica* isolates based on cgMLST. The circle size was proportional to the number of isolates. Clusters generated using the hierarchical clustering method from EnteroBase and using a 100 cgMLST allele distance (HC100) were represented by circles. **a** Isolates from this study. **b** Isolates from this study and the public database

An NJ tree and an MST based on cgMLST analysis of 187 *Y. enterocolitica* isolates were constructed. These 1,553 cgMLST target genes were randomly distributed across the genome. *Y. enterocolitica* isolates were divided into 125 cgMLST types (CTs) (Additional file 8: Table S6 and Fig. 6). CgMLST analysis revealed the core genome diversity of isolates with the same ST from 0 to 84 allelic differences. The NJ tree of 187 *Y. enterocolitica* isolates from Ningxia indicated the names of two microclades of the HC1490 (Hierarchical clustering) cluster (Fig. 6). HC1490_10 and HC1490_2 were the primary phylogenetic branches. These two microclades of the HC1490 cluster were consistent with the results of the *Y. enterocolitica* ML tree. HC1490_2 was strongly associated with biotypes 3, 4, and 5 isolates. HC1490_10 was closely related to biotype 1A isolates. The 125 CTs present in the 187 isolates from the Ningxia region clustered to form 54 microclades of HC100. Of these, HC100_2571 was the principal microclades, and the isolates were all from this study. The NJ tree constructed from isolates from the Ningxia region and those publicly available in the database showed that several microclades of the HC100 cluster were significantly associated with serotypes, hosts, and countries. HC100_2571 isolates were all obtained from this study and were of bioserotype 4/O:3 and 3/O:3, hosts were pigs and humans. HC100_406 isolates were 4/O:3, mostly from patients in New Zealand. The serotype of HC100_397 isolates was O:5,27, with hosts of pigs, humans, and food. HC100_2 and HC100_111 isolates were 3/O:9 and 4/O:9, respectively, mainly from pigs and humans in the UK and New Zealand. HC100_4570 isolates were biotype 5, obtained from sheep in Ningxia. HC100_466 isolates were biotype 1B, which were isolated from patients. HC100_150 isolates from pigs and patients in Ningxia were 1A/O:5. The isolates HC100_1273 were 1A/O:5 and obtained from pigs, cattle, and poultry in the Ningxia region. The mostly hosts of the biotype 1A isolates were pigs, cattle, sheep, rats, poultry, and food. The most common serotypes were O:5, O:8, O:9, and O:6,30 (Fig. 6 and Additional file 7: Fig. S7).

## Discussion

Ningxia Hui Autonomous Region is located in the upper reaches of the Yellow River in western China, where the largest Hui nationality in the country resides. Of the five cities in Ningxia, Guyuan and Wuzhong are Muslim-concentrated areas where livestock farms are not allowed to raise pigs, resulting in a small sample size in these two areas. Yinchuan and Zhongwei are areas with scattered Muslim populations, and 95.9% of the samples (259/270) in this study were collected from these two regions.

*Yersinia* presented a diversified epidemiological trend in Ningxia over a 12-year period, with a pattern of the diminished predominance of prevalent isolates and the coexistence of multiple subtypes. There is no significant upward or downward trend in detection rates between years. The detection rates of samples from different hosts were statistically different (*χ*^2^ = 22.636, *P* < 0.001), as well as 72.1% of poultry and livestock samples originated from pigs, implying that pigs were the primary source of *Yersinia* in Ningxia. *Y. enterocolitica* is present in the oral cavity (especially the tonsils and submaxillary lymph nodes), intestine, and feces of pigs [28, 29]. *Y. enterocolitica* was easily transmitted between pigs via the faecal-oral route and may also be spread through direct contact between humans and animals or the environment (water and soil) [30–32]. Therefore, people involved in swine farming and processing or those with livestock farms in the vicinity of their living area are at greater risk of *Y. enterocolitica* infection. Food (18.2%) was another major source of *Yersinia* infection in the Ningxia region, which is associated with the consumption of undercooked contaminated pork [29, 33]. It is potential for *Y. enterocolitica* to contaminate carcasses and pork products during the slaughter process [34]. Cross-contamination of other foods is possible when handling and preparing raw pork [35]. Because the transmission of *Y. enterocolitica* involves multiple pathways, the health department should continuously monitor and strengthen epidemiological investigation and training; the animal health department should improve quarantine and hygiene supervision of pigs and regulate the operation of workers in the breeding industry; the environment department should improve the supervision of breeding areas and their surrounding environment; the food regulatory department should continuously focus on and strengthen the testing of food with pig origin; the public should be concerned about food safety and avoid eating raw or undercooked meat products.

Public health and epidemiology face increasing challenges in understanding how environmental trends affect zoonotic disease dynamics [36]. An effective approach to zoonotic disease prevention and control that integrates animal, human, and environmental ecological principles is required [37]. The distribution of *Y. enterocolitica* was influenced by a variety of factors. Correlation analyses revealed that altitude, mean temperature and precipitation were important factors influencing the epidemiological diversity of *Y. enterocolitica* in animals in China, and were strongly related to climatic and agricultural characteristics [14]. It was evident that temperature and precipitation of the collection locations were closely related to the biotype, serotype, and pathogenicity of the isolates. The mean temperature and mean precipitation for pathogenic isolates were 12.70 °C and 1.34 mm, respectively, whereas the mean temperature and mean precipitation of non-pathogenic isolates were 16.85 °C and 2.54 mm, respectively, with the differences being highly statistically significant. This was because that *Y. enterocolitica* was a psychrotrophic bacteria that grew more slowly than most Gram-negative bacteria at room temperature and was unable to compete in mixed populations at high temperatures, but was able to grow, multiply and survive for long periods at low temperatures [38]. *Y. enterocolitica* must acclimate to the temperature of the human host before infection, therefore temperature-dependent chromosomal and plasmid-associated virulence determinants play an essential function [39]. In terms of precipitation, Ningxia has a typical continental semi-humid and semi-arid climate, with the rainy season lasting from June to September. Therefore we hypothesized that the significant differences in pathogenicity and precipitation were directly related to temperature. We used both unifactor and multifactor analysis in the prediction model, and it was obvious that the multifactor prediction result (temperature + precipitation + host) was the most optimal. Because neither humans nor microorganisms exist as independent individuals, various environment factors inevitably influence how organisms survive and contribute to the formation of specific ecological niches, necessitating a multifactorial approach. The random forest model outperformed the other three models in both the training and validation sets. This could benefit from the algorithmic advantages of the random forest, such as high performance and generalisation ability. Accuracy can be maintained even in the presence of missing data. Predictive models are powerful tools for public health and epidemiology, predicting the spread and spillover of pathogenic bacteria based on external environmental factors like temperature, precipitation, altitude, and vegetation distribution, as well as the characteristics of isolates like serotypes and biotypes, and monitoring and alerting on relevant zoonotic diseases.

Through robust and reproducible comparisons, WGS provides unprecedented resolution for characterising *Y. enterocolitica* [40], allowing it to be applied to public health management as well as real-time surveillance and outbreak investigation of foodborne diseases [41]. The ML tree of *Y. enterocolitica* isolates demonstrated that clustering was tightly linked to the biotype, which was consistent with previous findings [42, 43]. Biotype 3, 4, and 5 isolates displayed tight clusters with short terminal branches compared with biotype 1A isolates. This clade represented the earliest diverging branches of *Y. enterocolitica*, supporting the hypothesis that pathogenic members evolved from non-pathogenic ancestors [42]. Genome analysis reveals different evolutionary processes in pathogens that potentially influence their lifestyles and contribute to specific ecological niches. The typing results revealed regional differences in the distribution of STs and CTs, biotype 1A isolates contained 46 STs (85.2%, 46/54) and 73 (58.4%, 73/125) CTs, indicating a high degree of heterogeneity. Notably, isolates from patients in this study were classified into the same microclades as isolates from food and pigs, implying that *Y. enterocolitica* had the potential to infect humans via pigs and food. According to Karlsson et al. [44], an outbreak of *Y. enterocolitica* 4/O:3 in fresh prewashed spinach occurred in Sweden in 2019. This was completely consistent with the dominant bioserotype of the isolates in this study. Some studies have shown that this biological serotype is associated with approximately 80% of human infections of *Y. enterocolitica* worldwide [45]. Despite the fact that no *Yersinia* outbreaks have been reported in Ningxia, it is worthwhile to raise the alarm.

The distribution of virulence genes was closely related to biotype in the current study. The presence of the virulence factor for iron uptake is attributed to the high pathogenicity of biotype 1B isolates. Iron supply and production of the siderophore transport system were significant factors in the infection of *Y. enterocolitica* [46]. Except for biotype 1B isolates, other pathogenic strains utilise exogenous iron carriers such as ferroflavin B and ferroflavin E [47]. Different pathways of iron uptake may explain differences in the virulence among biotypes [48]. In comparison to biotype 1A isolates, biotypes 2, 3, 4, and 5 isolates had virulence genes associated with the invasion and T3SS. T3SS is primarily composed of multiple *Yersinia* outer proteins (Yops), low calcium response-stimulating proteins (LCRS), and heat-stable *enterocolitica* toxin (Yst) [49]. Gram-negative pathogens inject virulence proteins into the cytoplasm of target eukaryotic cells via Type III secretion system (T3SS) [50]. Biotype 1A strains are regarded as non-pathogenic. However, increasing epidemiological and experimental evidence indicates that biotype 1A strains can cause gastrointestinal disorders [51] and reactive arthritis [52] as well as being implicated in foodborne and nosocomial outbreaks around the world [53], implying a role for the *yst*B gene in the pathogenic process [54]. In the present research, 88.8% (n = 79) of the Biotype 1A isolates possessed the *yst*B gene. Although there was no evidence that biotype 1A isolates were pathogenic in this study, researchers should remain vigilant for non-pathogenic *Y. enterocolitica* as well.

There were several limitations to our study. Firstly, the patient sample size was small, and it was collected in 2018 and 2019. The reason is that symptom of diarrhoea caused by *Y. enterocolitica* are usually mild and self-limiting. Patients rarely visit outpatient clinics. Secondly, the collection of samples from both food and patient sources lacked quantitative homogeneity and temporal consistency. Therefore, statistical data and predictive models may be affected. Thirdly, we used ecological epidemiological study to explore the relationship between macro-level environmental factors and pathogenicity of *Y. enterocolitica*. Ecological fallacies in ecological studies are the most significant drawback of ecological studies and are difficult to avoid. Finally, there is a scarcity of epidemiologically relevant information on isolates from diarrhoea patients. These constraints emphasized the significance of the One Health approach. Future work in the human, animal, environmental, and other relevant sectors should be followed by the Tripartite Zoonoses Guide to optimally support countries and regions in addressing zoonotic diseases through a multisectoral, One Health approach [55]. Concrete measures such as early warning and early detection through the Global Early Warning System for health threats and emerging risks at the human-animal-ecosystems interface (GLEWS +), linking surveillance systems, establishing standardised detection methods, sharing laboratory resources, improving laboratory capacity, expanding epidemiology training, and conducting joint sectoral risk assessments [56, 57]. One Health offers a promising and valuable approach to successfully achieving optimal human and animal health while considering the protection of ecosystems and our shared environment.

## Conclusions

The distribution of STs and CTs was related to biotype, geographical area, serotype, and host. The distribution of virulence factors was closely related to the biotype. The distribution of pathogenic isolates was correlated to the environment factors of temperature and precipitation, and machine learning models predicted that the epic of *Y. enterocolitica* areas in the Ningxia region was concentrated in the north, northwest, and south. Our research integrated the ecological modeling and molecular epidemiology study which lead to the public health of *Y. enterocolitica.* It also served as a guide for epidemiological and public health analyses. Zoonotic diseases and other public safety threats can be better addressed using this approach, which addresses health threats at the human-animal-environment interface in a synergistic manner.

## Supplementary Information

**Additional file 1: Fig. S1** Differences in biotype of *Y. enterocolitica* isolates and ecological factors. a Temperature. b Precipitation. c Altitude. d NDVI.

**Additional file 2: Fig. S2** Differences in serotype of *Y. enterocolitica* isolates and ecological factors. a Temperature. b Precipitation. c Altitude. d NDVI.

**Additional file 3: Fig. S3** Differences in host of *Y. enterocolitica* isolates and ecological factors. a Temperature. b Precipitation. c Altitude. d NDVI.

**Additional file 4: Fig. S4** Variable importance of predictive model for pathogenicity of *Y. enterocolitica.* a GLM (General Linear Model). b RF (Random Forest model). c XGBOOST (eXtreme Gradient Boosting).

**Additional file 5: Fig. S5** The risk of pathogenic of *Yersinia enterocolitica* by machine learning model predictions in study regions. a‒c The RF model predict the probability of pathogenic of *Y. enterocolitica* with sample source from animal, food, and human, respectively. d‒f The GLM model predict the probability of pathogenic of *Y. enterocolitica* with sample source from animal, food, and human, respectively. g‒i The XGBOOST model predict the probability of pathogenic of *Y. enterocolitica* with sample source from animal, food, and human, respectively. The ecological variables of temperature and precipitation were based on the monthly mean value of Ningxia region on August 2019.

**Additional file 6: Fig. S6** The Minimum Spanning Trees (MST) of *Y. enterocolitica* isolates. The circle size was proportional to the number of isolates. Links between circles were represented according to the number of allelic differences between STs. a Biotype. b Serotype.

**Additional file 7: Fig. S7** The Neighbor-joining (NJ) tree of *Y. enterocolitica* isolates based on cgMLST. a Biotype. b Serotype. c Host. d Country.

**Additional file 8: Table S1.** Summary of environment and climatic data sources. **Table S2.** List of the *Yersinia *reference genomes. **Table S3.** List of 88 *Y.enterocolitica *strains downloaded from the NCBI database. **Table S4.** Analysis results of training and test datasets. **Table S5.** List of 270 *Yersinia *isolates in the Ningxia Hui Autonomous Region** Table S6.** Origin, serotype, STs, and CTs of 187 strains of *Y. enterocolitica*.

## Data Availability

FASTQ datafiles have been deposited in NCBI-SRA and public archives under the project’s accession number PRJNA993921 (Centers for Disease Control and Prevention). The accession numbers of all isolates are provided in Additional file 8: Table S5. All data generated or analyzed during this study are included in this published article. The Additional information files are freely available to any scientist wishing to use them for non-commercial purposes upon request via e-mail with the corresponding author.

## Abbreviations

ANI: Average nucleotide identity
SNP: Single nucleotide polymorphism
cgMLST: Core gene multilocus sequence typing
RF: Random forest
STs: Sequence types
CTs: CgMLST types
NDVI: Normalized difference vegetation index
ML: Maximum likelihood
VFDB: Virulence factor database
HeirCC: Hierarchical cgMLST clustering
MST: Minimum spanning tree
NJ: Neighbor-joining
GLM: General linear model
RF: Random forest model
XGBOOST: eXtreme gradient boosting
ROC: Receiver operating characteristic
AUC: Area under the curves
BAPS: Bayesian analysis of population structure
HC: Hierarchical clustering
Yops: *Yersinia* outer proteins
LCRS: Low calcium response-stimulating proteins
Yst: Heat-stable *enterocolitica* toxin
T3SS: Type III secretion system
GLEWS+: The Global Early Warning System for health threats and emerging risks at the human–animal–ecosystems interface
