# Supplementary figures and images for: Public health implications of Yersinia enterocolitica investigation: an ecological modeling and molecular epidemiology study
Source: Infect Dis Poverty. 2023 Apr 21;12:41. doi: 10.1186/s40249-023-01063-6 (PMC10120104; doi:10.1186/s40249-023-01063-6)

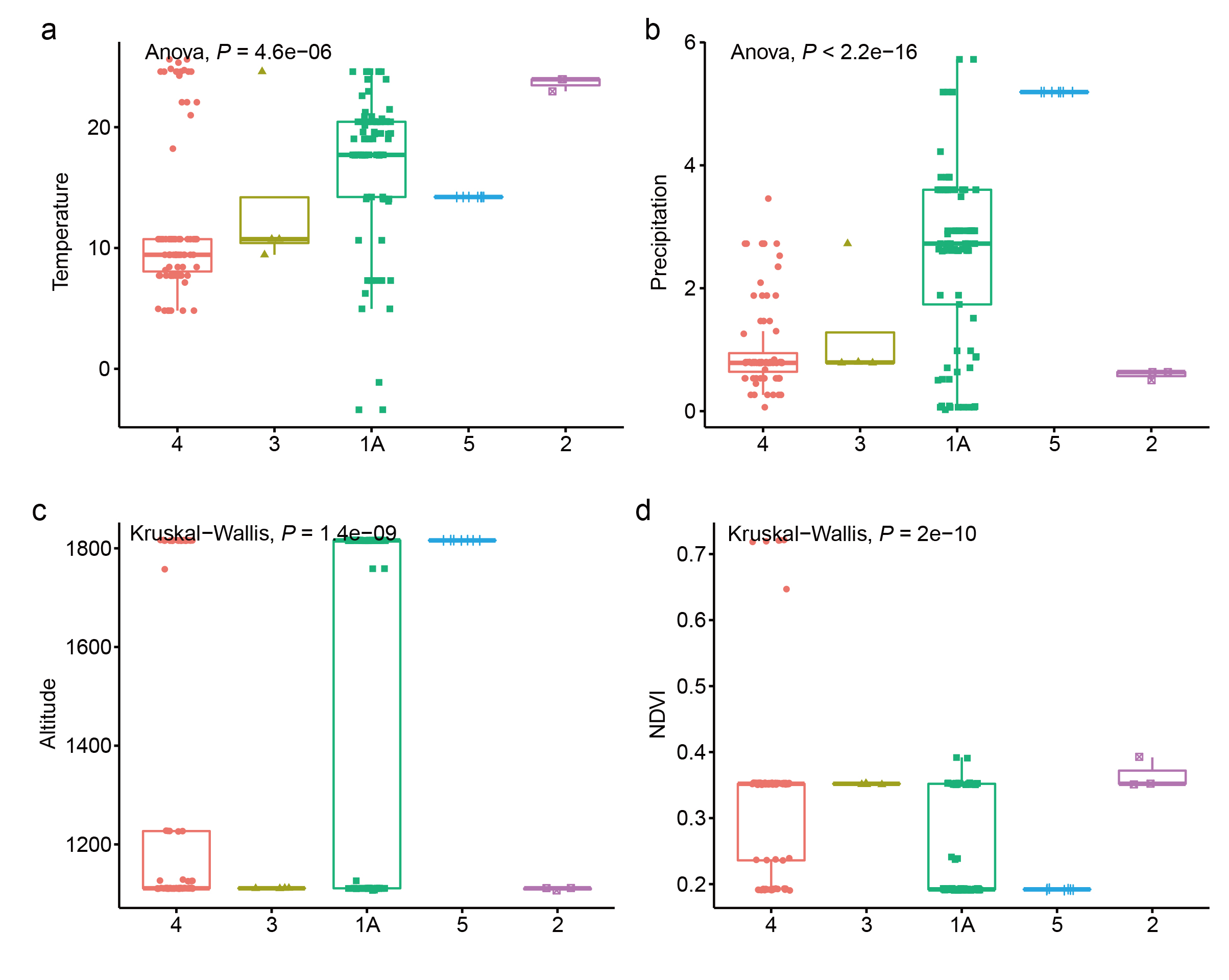

Supplement: Supplementary file 1 — Additional file 1: Fig. S1 Differences in biotype of Y. enterocolitica isolates and ecological factors. a Temperature. b Precipitation. c Altitude. d NDVI. [file 40249_2023_1063_MOESM1_ESM.jpg]

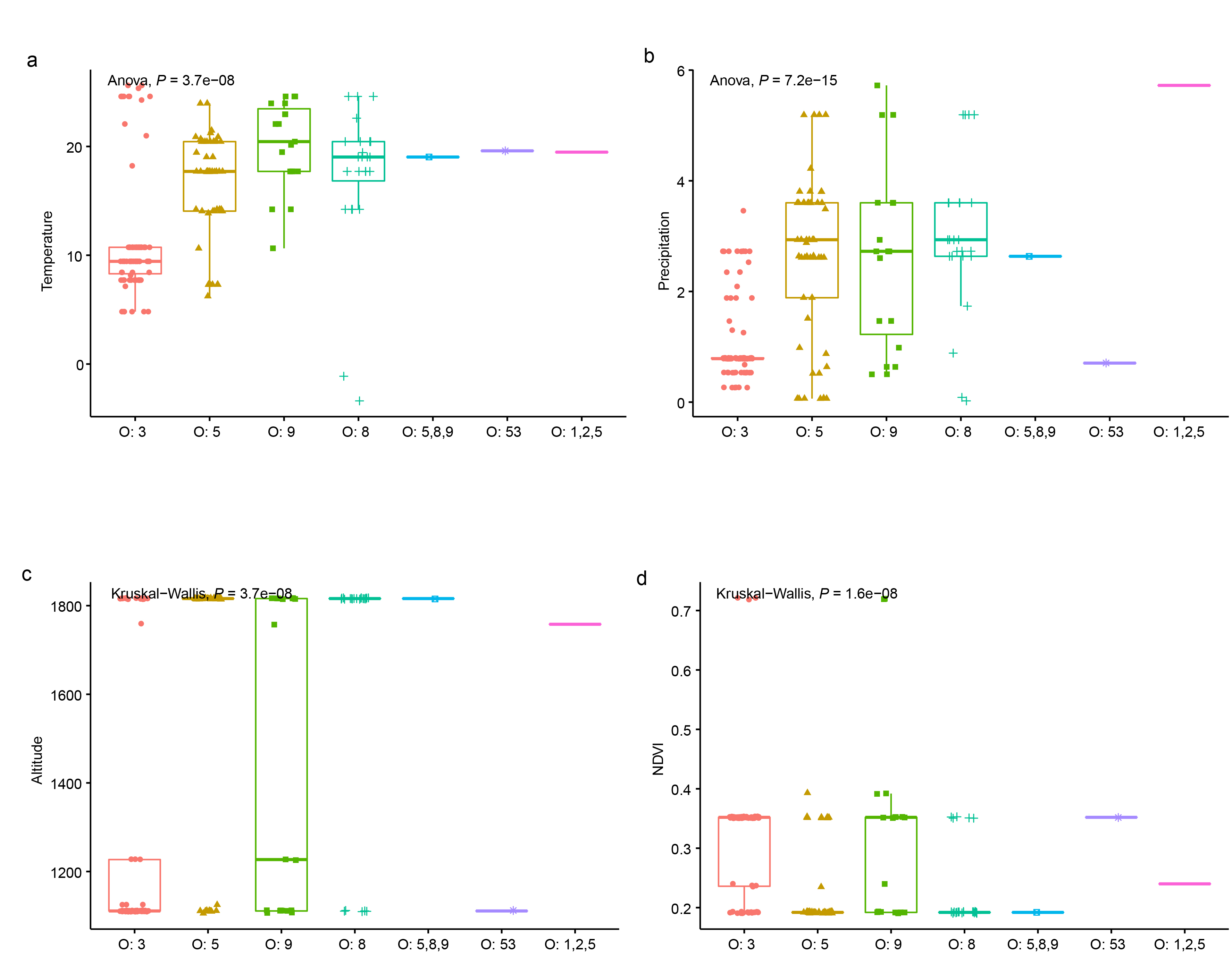

Supplement: Supplementary file 2 — Additional file 2: Fig. S2 Differences in serotype of Y. enterocolitica isolates and ecological factors. a Temperature. b Precipitation. c Altitude. d NDVI. [file 40249_2023_1063_MOESM2_ESM.jpg]

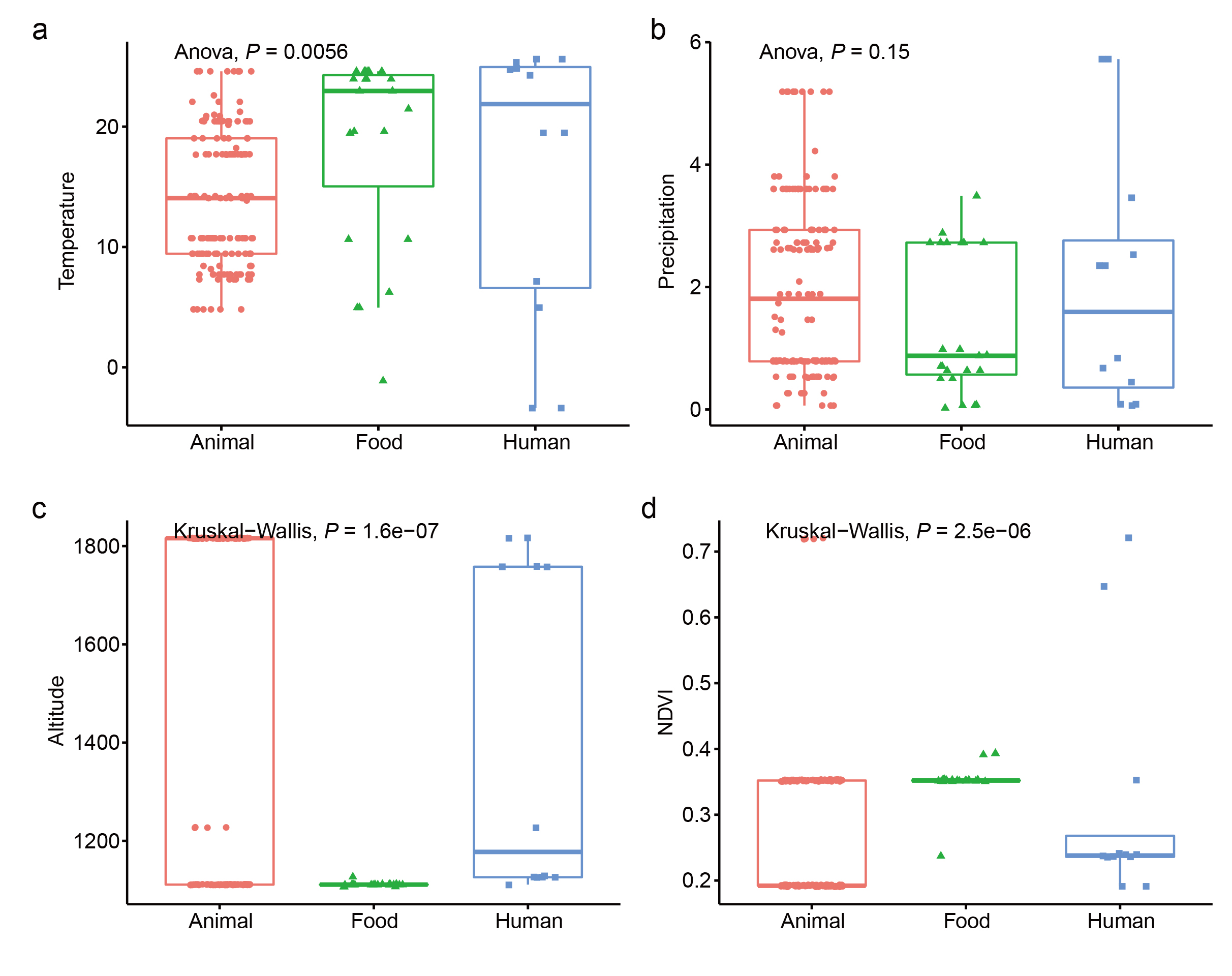

Supplement: Supplementary file 3 — Additional file 3: Fig. S3 Differences in host of Y. enterocolitica isolates and ecological factors. a Temperature. b Precipitation. c Altitude. d NDVI. [file 40249_2023_1063_MOESM3_ESM.jpg]

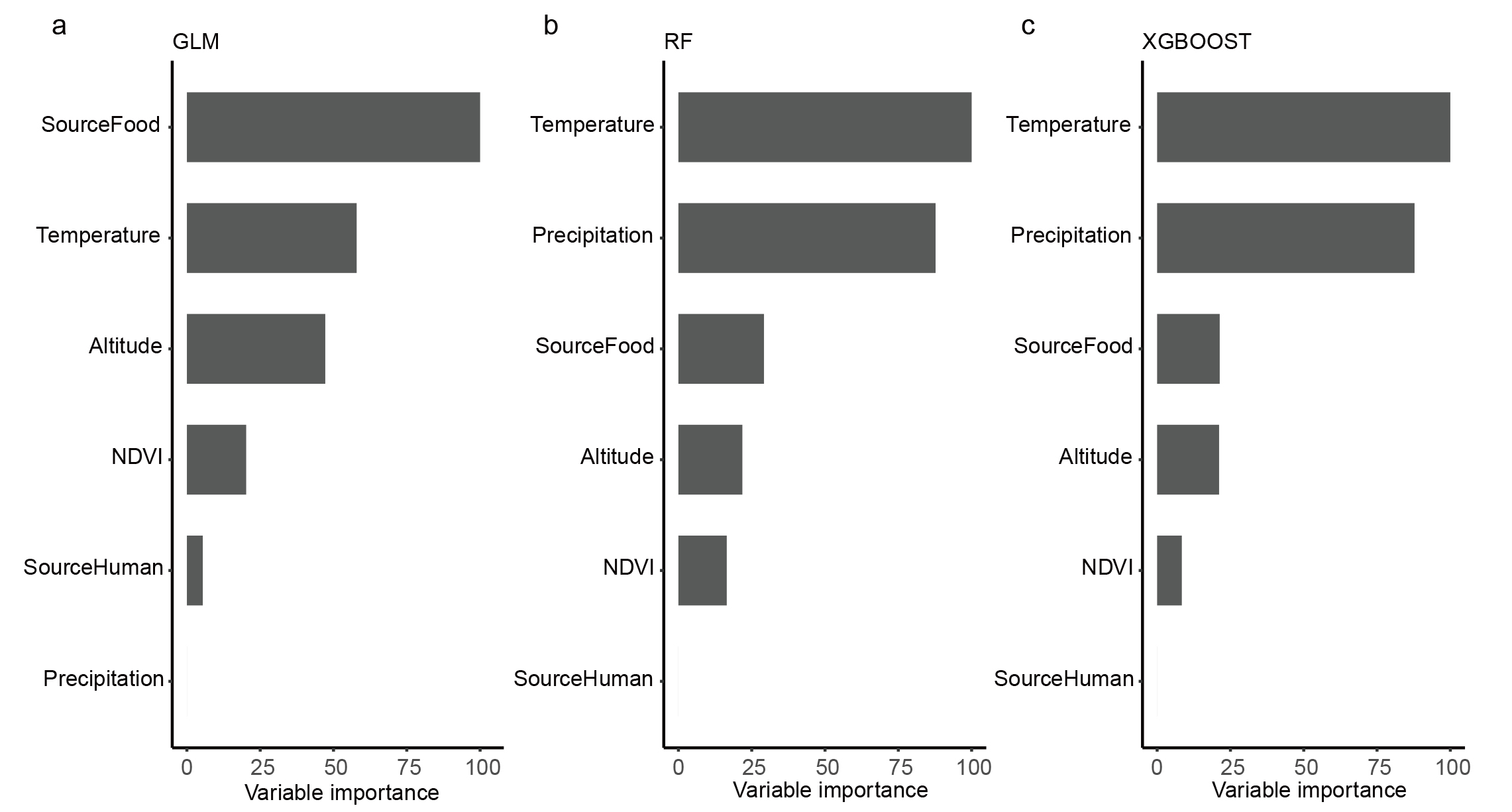

Supplement: Supplementary file 4 — Additional file 4: Fig. S4 Variable importance of predictive model for pathogenicity of Y. enterocolitica. a GLM (General Linear Model). b RF (Random Forest model). c XGBOOST (eXtreme Gradient Boosting). [file 40249_2023_1063_MOESM4_ESM.jpg]

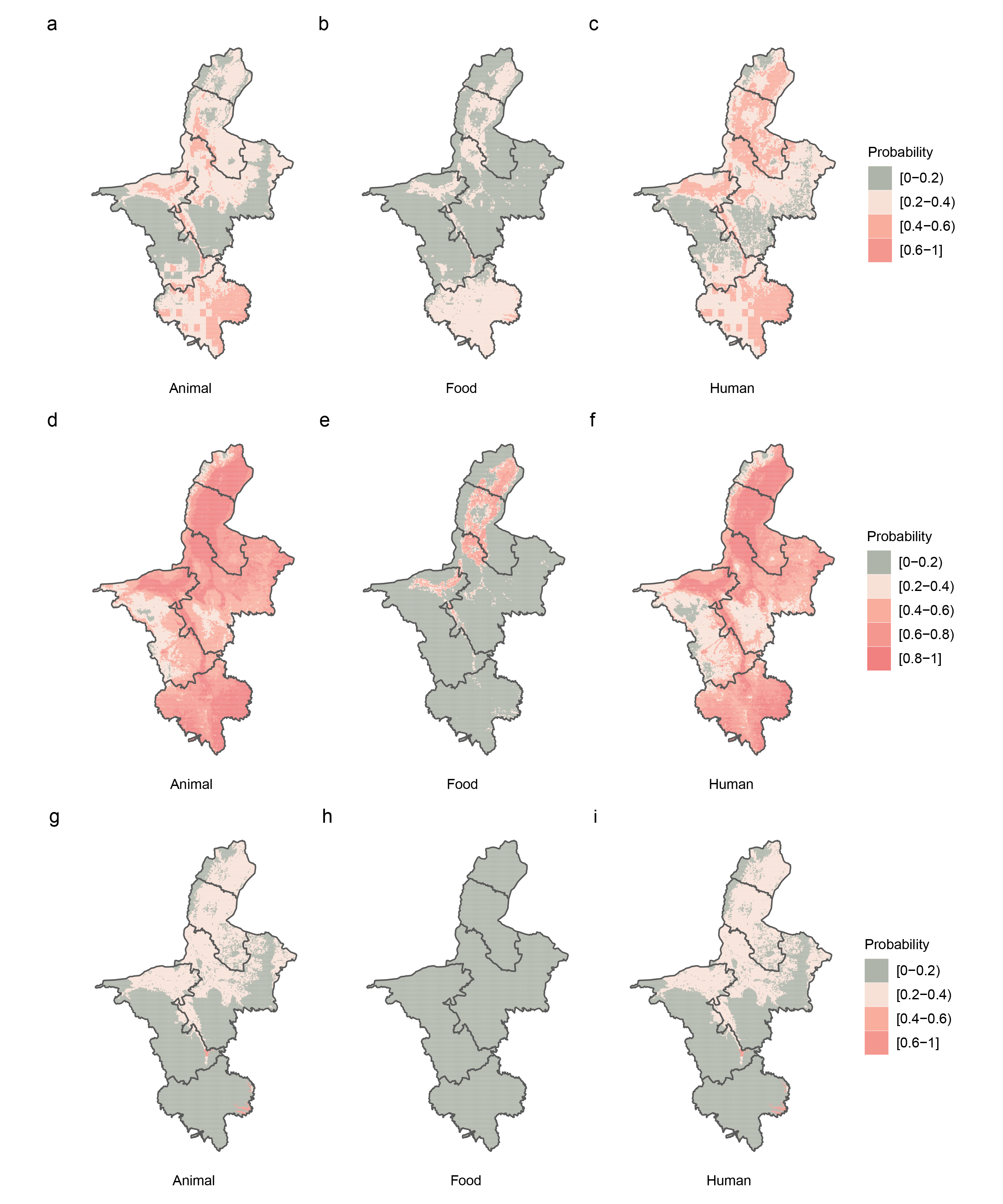

Supplement: Supplementary file 5 — Additional file 5: Fig. S5 The risk of pathogenic of Yersinia enterocolitica by machine learning model predictions in study regions. a‒c The RF model predict the probability of pathogenic of Y. enterocolitica with sample source from animal, food, and human, respectively. d‒f The GLM model predict the probability of pathogenic of Y. enterocolitica with sample source from animal, food, and human, respectively. g‒i The XGBOOST model predict the probability of pathogenic of Y. enterocolitica with sample source from animal, food, and human, respectively. The ecological variables of temperature and precipitation were based on the monthly mean value of Ningxia region on August 2019. [file 40249_2023_1063_MOESM5_ESM.jpg]

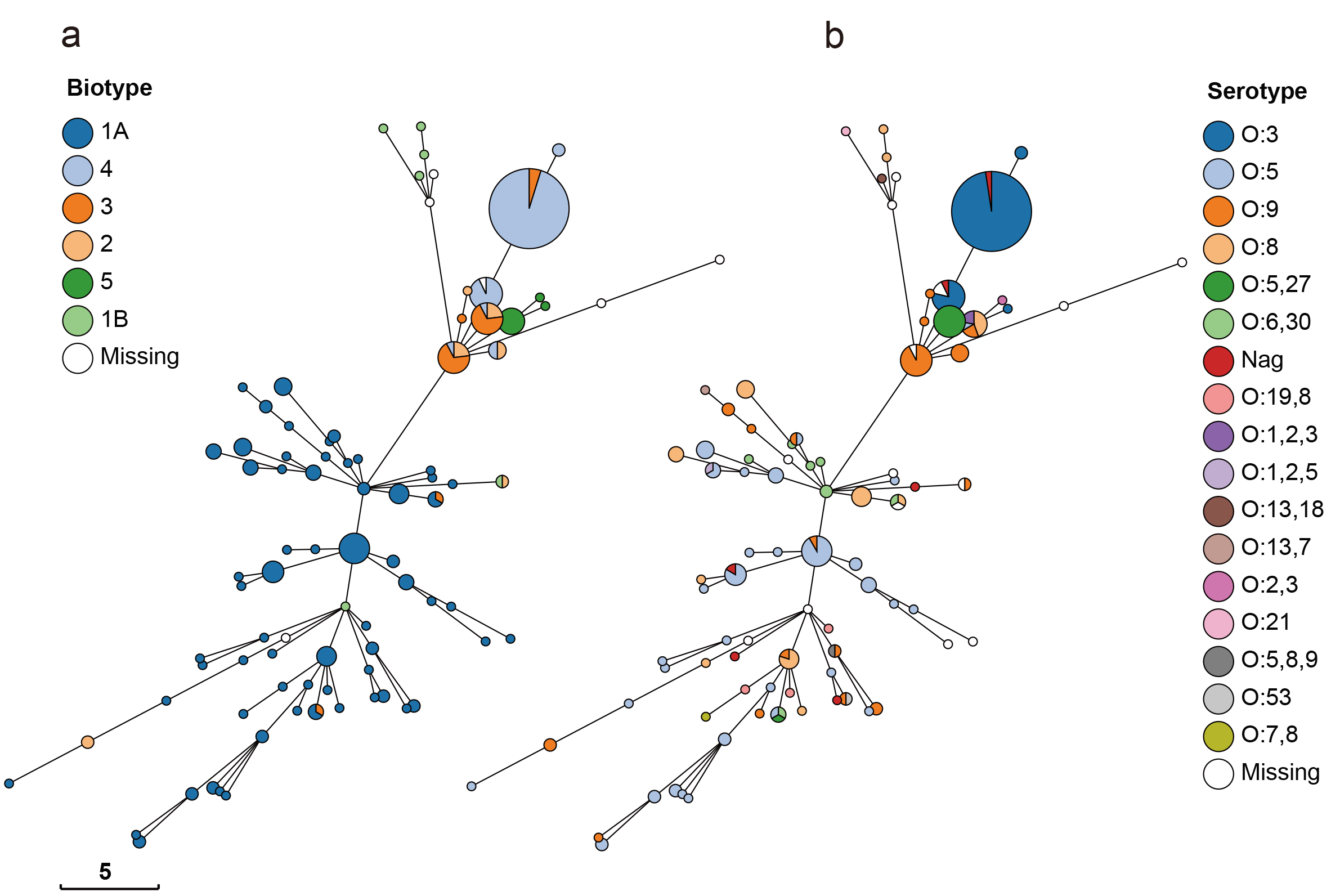

Supplement: Supplementary file 6 — Additional file 6: Fig. S6 The Minimum Spanning Trees (MST) of Y. enterocolitica isolates. The circle size was proportional to the number of isolates. Links between circles were represented according to the number of allelic differences between STs. a Biotype. b Serotype. [file 40249_2023_1063_MOESM6_ESM.jpg]

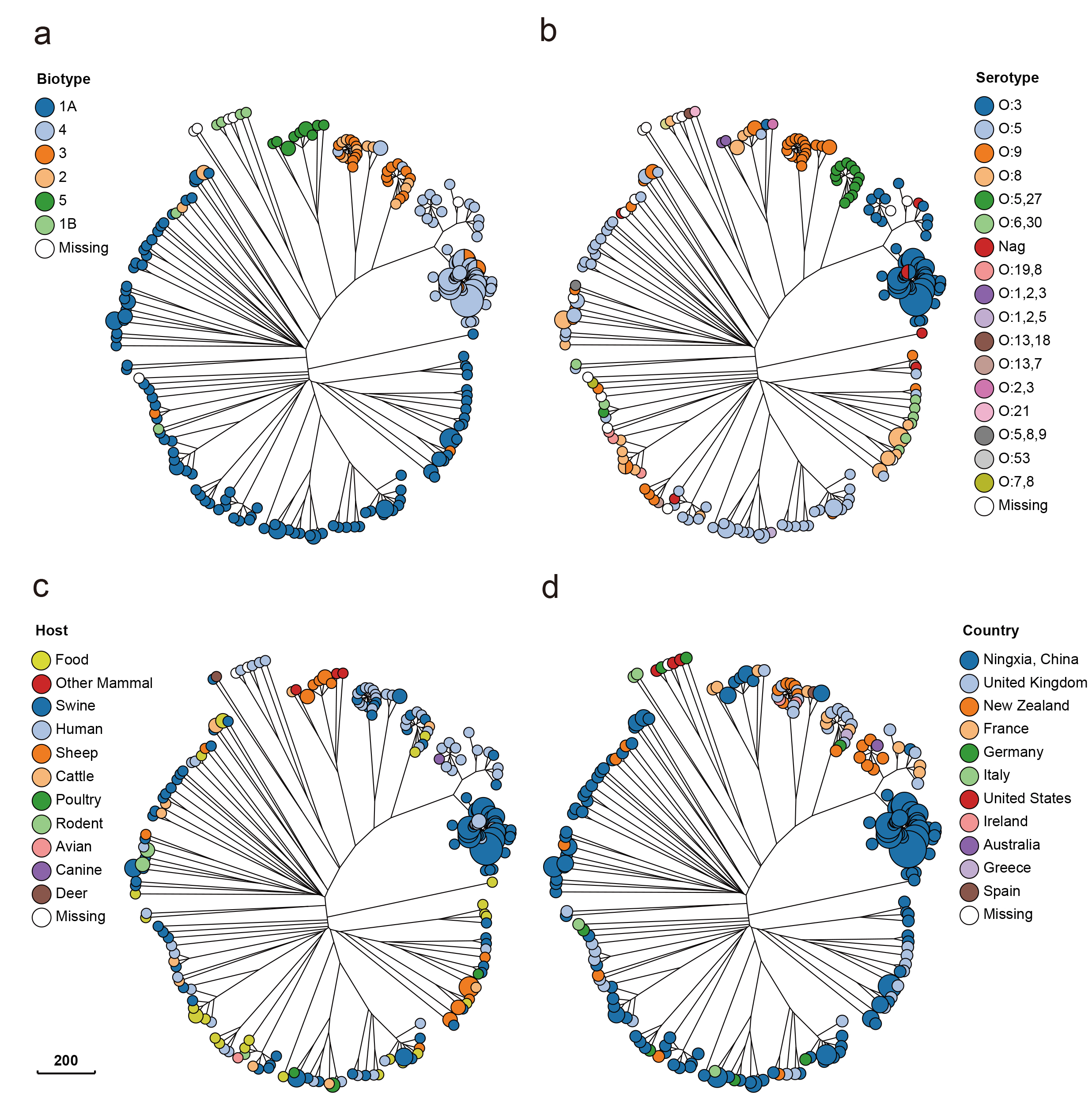

Supplement: Supplementary file 7 — Additional file 7: Fig. S7 The Neighbor-joining (NJ) tree of Y. enterocolitica isolates based on cgMLST. a Biotype. b Serotype. c Host. d Country. [file 40249_2023_1063_MOESM7_ESM.jpg]
