# Supplementary material for: Public health implications of Yersinia enterocolitica investigation: an ecological modeling and molecular epidemiology study
Source: Infect Dis Poverty. 2023 Apr 21;12:41. doi: 10.1186/s40249-023-01063-6 (PMC10120104; doi:10.1186/s40249-023-01063-6)
Supplement: Supplementary file 8 — Additional file 8: Table S1. Summary of environment and climatic data sources. Table S2. List of the Yersinia reference genomes. Table S3. List of 88 Y.enterocolitica strains downloaded from the NCBI database. Table S4. Analysis results of training and test datasets. Table S5. List of 270 Yersinia isolates in the Ningxia Hui Autonomous Region Table S6. Origin, serotype, STs, and CTs of 187 strains of Y. enterocolitica. [file 40249_2023_1063_MOESM8_ESM.docx]

**Table S1** Summary of environment and climatic data sources

| Data Type | Source | Period |
| --- | --- | --- |
| Monthly mean temperature | http://modis.gsfc.nasa.gov/. | 2007-2019 |
| Monthly mean rainfall | http://modis.gsfc.nasa.gov/. | 2007-2019 |
| Elevation | http://data.cma.cn/ | 2010 |
| NDVI^a^ | http://modis.gsfc.nasa.gov/. | 2020 |

^a^normalized difference vegetation index

**Table S2** List of the *Yersinia* reference genomes

| Specises | GenBank/RefSeq assembly accession |
| --- | --- |
| *Y. enterocolitica* | GCA_000009345.1 |
| *Y. intermedia* | GCF_009730055.1 |
| *Y. massiliensis* | GCF_000312485.1 |
| *Y. mollaretii* | GCF_013282725.1 |
| *Y. frederiksenii* | GCF_000754805.1 |
| *Y. kristensenii* | GCA_900460525.1 |
| *Y. hibernica* | GCF_004124235.1 |
| *Y. canariae* | GCF_009831415.1 |
| *Y. rochesterensis* | GCF_003600645.1 |
| *Y. nurmii* | GCF_001112925.1 |
| *Y. pestis* | GCF_000222975.1 |
| *Y. ruckeri* | GCF_017498685.1 |
| *Y. aldovae* | GCA_000834395.1 |
| *Y. aleksiciae* | GCF_001319845.1 |
| *Y. bercovieri* | GCA_001319545.1 |
| *Y. entomophaga* | GCF_001656035.1 |
| *Y. pekkanenii* | GCF_001152565.1 |
| *Y. pseudotuberculosis* | GCA_900637475.1 |
| *Y. rohdei* | GCF_000834455.1 |
| *Y. wautersii* | GCA_001319825.1 |
| *Y. thracica* | GCF_902170565.1 |
| *Y. artesiana* | GCF_902726545.1 |
| *Y. vastinensis* | GCF_902726565.1 |
| *Y. alsatica* | GCF_902170305.1 |
| *Y. proxima* | GCA_902170785.1 |
| *Y. similis* | GCA_000582515.1 |

**Table S3** List of 88 *Y.enterocolitica* strains downloaded from the NCBI database.

| SRA | Collection Date | Origin | Biotype | Serotype | Geographic Location | STs | CTs |
| --- | --- | --- | --- | --- | --- | --- | --- |
| ERR024541 | 2002 | Pig | 3 | O: 5,27 | United Kingdom | 14 | 3370 |
| ERR024542 | 2003 | Human | 3 | O: 9 | United Kingdom | 12 | 3336 |
| ERR024546 | 2002 | Cattle | 1A | O: 6, 30 | United Kingdom | 16 | 3332 |
| ERR024549 | 2003 | Human | 3 | O: 9 | United Kingdom | 12 | 3195 |
| ERR024550 | 2003 | Human | 3 | O: 5,27 | United Kingdom | 14 | 3333 |
| ERR024551 | 2002 | Pig | 1A | O: 6,30 | United Kingdom | 4 | 3179 |
| ERR024554 | 2003 | Human | 1A | O: 19, 8 | United Kingdom | 573 | 3183 |
| ERR024555 | 2002 | Pig | NK | O: 9 | United Kingdom | 12 | 3397 |
| ERR024557 | 2008 | Pig | 3 | O: 9 | United Kingdom | 12 | 3192 |
| ERR024558 | 2002 | Sheep | 3* | O: 6, 30 | United Kingdom | 5 | 3360 |
| ERR024560 | 2002 | Pig | 3 | O: 5,27 | United Kingdom | 14 | 3376 |
| ERR024562 | 2002 | Pig | 1A | NK | United Kingdom | 5 | 3212 |
| ERR024563 | 2003 | Human | 1A | O: 6, 30 | United Kingdom | 7 | 3354 |
| ERR024566 | 2002 | Pig | 4 | O: 3 | United Kingdom | 18 | 3178 |
| ERR024567 | 2008 | Human | 3 | O: 9 | New Zealand | 571 | 3177 |
| ERR024569 | 2005 | Avian | 1A | NK | New Zealand | 20 | 3306 |
| ERR024570 | 2003 | Human | 4 | O: 3 | United Kingdom | 18 | 3343 |
| ERR024571 | 2003 | Human | 1A | O: 5 | United Kingdom | 8 | 3208 |
| ERR024573 | 2007 | Sheep | 1A | NK | New Zealand | 147 | 4151 |
| ERR024574 | 2008 | Human | 2 | O: 9 | New Zealand | 572 | 3165 |
| ERR024575 | 2002 | Dog | 4 | O: 3 | New Zealand | 18 | 3276 |
| ERR024577 | 2008 | Human | 3 | O: 9 | New Zealand | 12 | 3261 |
| ERR024579 | 2007 | Human | 4 | O: 3 | New Zealand | 18 | 3273 |
| ERR024580 | 2008 | Human | 4 | O: 3 | New Zealand | 18 | 3318 |
| ERR024581 | 2008 | Human | 3 | O: 9 | New Zealand | 12 | 3425 |
| ERR024583 | 2002 | Pig | 3 | O: 9 | United Kingdom | 12 | 3349 |
| ERR024584 | 2002 | Pig | 4 | O: 3 | United Kingdom | 18 | 3381 |
| ERR024585 | 2002 | Pig | 1A | O: 5 | United Kingdom | 3 | 3190 |
| ERR024586 | 2006 | Human | 1B* | Nag | New Zealand | 26 | 3418 |
| ERR024587 | 2003 | Food | 3 | O: 5,27 | New Zealand | 14 | 3148 |
| ERR024588 | 2008 | Human | 1A | NK | New Zealand | 11 | 3259 |
| ERR024589 | 2003 | Human | 2 | O: 9 | New Zealand | 12 | 3254 |
| ERR024590 | 2002 | Human | 1B* | NK | New Zealand | 2 | 3422 |
| ERR024597 | 1989 | Human | 3 | O: 5,27 | Germany | 14 | 3428 |
| ERR024599 | 1997 | Human | 1A | O: 5 | Germany | 166 | 4140 |
| ERR024601 | 1984 | Human | 1B | O: 21 | Germany | 196 | 3166 |
| ERR024602 | 2008 | Pig | 1A | O: 7, 8 | Germany | 190 | 3163 |
| ERR024856 | 2002 | Cattle | 1A | O: 19,8 | United Kingdom | -165 | 4187 |
| ERR024857 | 2002 | Cattle | 3 | O: 5,27 | United Kingdom | 14 | 3335 |
| ERR024858 | 2002 | Human | 1A | NK | United Kingdom | 184 | 3329 |
| ERR024859 | 2002 | Cattle | 3* | O: 5,27 | United Kingdom | 19 | 3303 |
| SRA | Collection Date | Origin | Biotype | Serotype | Geographic Location | STs | CTs |
| ERR024860 | 2002 | Pig | 3 | O: 5,27 | United Kingdom | 14 | 3346 |
| ERR024861 | 2003 | Pig | 1A | O: 19,8 | United Kingdom | 197 | 3348 |
| ERR024862 | 2003 | Human | 1A | O: 6,30 | United Kingdom | 19 | 3340 |
| ERR024863 | 2003 | Human | 4 | O: 3 | New Zealand | 18 | 3267 |
| ERR024864 | 2008 | Human | 4 | O: 3 | New Zealand | 18 | 4139 |
| ERR024865 | 2005 | Human | 1A | O: 5 | New Zealand | 578 | 3248 |
| ERR024866 | 2002 | Pig | 2 | O: 9 | United Kingdom | 12 | 3404 |
| ERR024867 | 2006 | Human | 4 | ND | New Zealand | 18 | 3173 |
| ERR024869 | 2002 | Pig | 3 | O: 9 | United Kingdom | 12 | 3352 |
| ERR024871 | 2003 | Human | 3 | O: 5,27 | United Kingdom | 14 | 3350 |
| ERR024873 | 2002 | Pig | 1A | O: 6, 30 | United Kingdom | 4 | 3345 |
| ERR024874 | 2007 | Human | 1A | O: 13,7 | Germany | 191 | 3271 |
| ERR024875 | 2002 | Sheep | 1A | O: 6, 30 | United Kingdom | 123 | 3342 |
| ERR024876 | 2002 | Pig | 3 | O: 9 | United Kingdom | 12 | 3358 |
| ERR024877 | 2002 | Pig | 3 | O: 5,27 | United Kingdom | 14 | 3327 |
| ERR024879 | 2003 | Human | 1A | O: 6, 30 | United Kingdom | 17 | 3353 |
| ERR024880 | 2003 | Human | 1A | O: 5 | United Kingdom | 3 | 3355 |
| ERR024884 | 2004 | Human | 1B | O: 8 | Germany | 368 | 4141 |
| ERR024886 | 1993 | Human | 1A | O: 5 | Germany | 3 | 3282 |
| ERR024887 | 2008 | NK | 1B | O: 8 | United States | 176 | 3298 |
| ERR024889 | 2004 | Human | 1B | O: 8 | Germany | 579 | 3300 |
| ERR027406 | 2008 | Pig | 3 | NK | Ireland | 12 | 3288 |
| ERR027419 | 2008 | Human | 3 | O: 9 | Ireland | 12 | 3253 |
| ERR163896 | 1998 | Food | 1A | O: 5 | Italy | 173 | 3287 |
| ERR163898 | 1988 | Food | 2 | O: 5,27 | Greece | 14 | 3156 |
| ERR163899 | 1998 | Human | 2 | O: 5,27 | France | 14 | 3442 |
| ERR163900 | 1989 | Human | 2 | O: 9 | Spain | 145 | 3269 |
| ERR163901 | 1990 | Pig | 2 | O: 9 | United Kingdom | 12 | 3334 |
| ERR163902 | 2000 | Human | 2 | O: 9 | France | 145 | 3262 |
| ERR163903 | 2007 | Human | 3 | O: 5,27 | France | 14 | 3250 |
| ERR163904 | 2007 | Human | 3 | O: 5,27 | France | 14 | 3264 |
| ERR163905 | 1982 | Human | 4 | O: 3 | France | 18 | 3443 |
| ERR163906 | 1991 | Human | 4 | O: 3 | Australia | 18 | 3274 |
| ERR163907 | 1999 | Human | 4 | O: 3 | France | 18 | 4160 |
| ERR163908 | 2008 | Human | 4 | O: 3 | France | 18 | 3283 |
| ERR163909 | 2008 | Human | 1B | O: 8 | Belgium | 188 | 3246 |
| ERR163913 | 2008 | Human | 1B | O: 13,18 | United States | 195 | 3270 |
| ERR163915 | 2008 | Cattle | 5 | O: 1,2,3 | France | 13 | 3439 |
| ERR163916 | 2008 | Hare | 5 | O: 2,3 | United Kingdom | 174 | 3200 |
| ERR163919 | 2008 | Hare | 5 | O: 1,2,3 | France | 13 | 3308 |
| ERR163920 | 2008 | Hare | 5 | O: 3 | France | 187 | 3155 |
| SRR2149856 | NK | Human | 1B | O: 8 | United States | 1 | 73 |
| SRA | Collection Date | Origin | Biotype | Serotype | Geographic Location | STs | CTs |
| SRR2180266 | 2015 | Human | NK | NK | United States | 154 | 3134 |
| SRR5154577 | 2014 | Wild boar | 1B | O: 1,2,3,5,8,9 | Italy | 235 | 2260 |
| SRR5154578 | 2014 | Wild boar | 1A | NK | Italy | 236 | 1863 |
| SRR5154580 | 2014 | Wild boar | 1A | NK | Italy | 237 | 2259 |
| LC20 | 2008 | Rat | NK | NK | Zhejiang, China | NK | NK |
| 105.5R | NK | Human | 3 | O:9 | Liaoning, China | NK | NK |

Note: STs, sequences types; CTs, cgMLST types; NA, not applicable; Nag, nonagglutinative; NK, not known.*Inconsistency in typing vs. phylogenetic grouping.

**Table S4** Analysis results of training and test datasets

|  |  | Test (*n* = 35) | Train (*n* = 152) | *P* |
| --- | --- | --- | --- | --- |
| Serotype | Nag | 1 (2.9%) | 6 (4.0%) | 0.851 |
|  | O: 1,2,5 | 0 (0.00%) | 1 (0.7%) |  |
|  | O: 3 | 18 (51.4%) | 65 (42.8%) |  |
|  | O: 5 | 8 (22.9%) | 43 (28.3%) |  |
|  | O: 5,8,9 | 0 (0.00%) | 1 (0.7%) |  |
|  | O: 53 | 0 (0.00%) | 1 (0.7%) |  |
|  | O: 8 | 6 (17.1%) | 18 (11.8%) |  |
|  | O: 9 | 2 (5.7%) | 17 (11.2%) |  |
| Biotype | 1A | 14 (40.0%) | 75 (49.3%) | 0.554 |
|  | 2 | 0 (0.00%) | 3 (2.0%) |  |
|  | 3 | 0 (0.00%) | 4 (2.6%) |  |
|  | 4 | 19 (54.3%) | 65 (42.8%) |  |
|  | 5 | 2 (5.7%) | 5 (3.3%) |  |
| Pathogenicity | pathogenic | 21 (60.0%) | 77 (50.7%) | 0.418 |
|  | Non-pathogenic | 14 (40.0%) | 75 (49.3%) |  |
| Temperature |  | 13.1 (6.5) | 15.0 (6.5) | 0.112 |
| Precipitation |  | 1.88 (1.5) | 1.92 (1.5) | 0.872 |
| Altitude |  | 1457 (354) | 1457 (350) | 0.993 |
| NDVI |  | 0.28 (0.1) | 0.28 (0.1) | 0.887 |

Note: Data of serotype, biotype, and pathogenicity indicated the number and percentage of isolates. Data of temperature, precipitation, elevation, and NDVI indicated mean and standard deviation.

**Table S5** List of 270 *Yersinia* isolates in the Ningxia Hui Autonomous Region

| BioSample | Samplename | Collection Date | Origin | Biochemical tests* | ANI analysis | Serotype | Location | [Coordinate](C:/Users/%E6%9C%88%E5%84%BF/AppData/Local/youdao/dict/Application/8.9.6.0/resultui/html/index.html#/javascript:;) |
| --- | --- | --- | --- | --- | --- | --- | --- | --- |
| SAMN19699696 | NX0738 | 2007-10-23 | Pig | 0014520 (95%) | *Y.enterocolitica* | O: 3 | Yinchuan | 38.47 N 106.27 E |
| SAMN19699697 | NX0740 | 2007-10-23 | Pig | 0014520 (95%) | *Y.enterocolitica* | O: 3 | Yinchuan | 38.47 N 106.27 E |
| SAMN19699698 | NX0741 | 2007-10-23 | Pig | 0014520 (95%) | *Y.enterocolitica* | O: 3 | Yinchuan | 38.47 N 106.27 E |
| SAMN19699699 | NX0742 | 2007-10-23 | Pig | 0014520 (95%) | *Y.enterocolitica* | O: 3 | Yinchuan | 38.47 N 106.27 E |
| SAMN19699700 | NX0743 | 2007-10-23 | Pig | 0014520 (95%) | *Y.enterocolitica* | O: 3 | Yinchuan | 38.47 N 106.27 E |
| SAMN19699701 | NX0744 | 2007-10-23 | Pig | 0014520 (95%) | *Y.enterocolitica* | O: 3 | Yinchuan | 38.47 N 106.27 E |
| SAMN19699702 | NX0745 | 2007-10-23 | Pig | 0014520 (95%) | *Y.enterocolitica* | O: 3 | Yinchuan | 38.47 N 106.27 E |
| SAMN19699703 | NX0746 | 2007-10-23 | Pig | 0014520 (95%) | *Y.enterocolitica* | O: 3 | Yinchuan | 38.47 N 106.27 E |
| SAMN19699704 | NX0747 | 2007-10-23 | Pig | 0014520 (95%) | *Y.enterocolitica* | O: 3 | Yinchuan | 38.47 N 106.27 E |
| SAMN19699705 | NX0748 | 2007-10-23 | Pig | 0014520 (95%) | *Y.enterocolitica* | O: 3 | Yinchuan | 38.47 N 106.27 E |
| SAMN19699706 | NX0749 | 2007-10-23 | Pig | 0014520 (95%) | *Y.enterocolitica* | O: 3 | Yinchuan | 38.47 N 106.27 E |
| SAMN19699707 | NX0750 | 2007-10-23 | Pig | 0014520 (95%) | *Y.enterocolitica* | O: 3 | Yinchuan | 38.47 N 106.27 E |
| SAMN19699708 | NX0751 | 2007-10-23 | Pig | 0014520 (95%) | *Y.enterocolitica* | O: 3 | Yinchuan | 38.47 N 106.27 E |
| SAMN19699709 | NX0752 | 2007-10-23 | Pig | 0014520 (95%) | *Y.enterocolitica* | O: 3 | Yinchuan | 38.47 N 106.27 E |
| SAMN19699710 | NX0753 | 2007-10-23 | Pig | 0014520 (95%) | *Y.enterocolitica* | O: 3 | Yinchuan | 38.47 N 106.27 E |
| SAMN19699711 | NX0754 | 2007-10-23 | Pig | 0014520 (95%) | *Y.enterocolitica* | O: 3 | Yinchuan | 38.47 N 106.27 E |
| SAMN19699712 | NX0755 | 2007-10-23 | Pig | 0014520 (95%) | *Y.enterocolitica* | O: 3 | Yinchuan | 38.47 N 106.27 E |
| SAMN19699713 | NX0756 | 2007-10-23 | Pig | 0014520 (95%) | *Y.enterocolitica* | O: 3 | Yinchuan | 38.47 N 106.27 E |
| SAMN19699714 | NX08020 | 2008-07-06 | Pig | 0014520 (95%) | *Y.enterocolitica* | O: 3 | Yinchuan | 38.47 N 106.27 E |
| SAMN19699715 | NX08021 | 2008-07-06 | Pig | 1054500 (92.5%) | *Y.hibernica* | NA | Zhongwei | 36.56 N 105.64 E |
| SAMN19699716 | NX09001 | 2009-03-28 | Pig | 0014520 (95%) | *Y.enterocolitica* | O: 3 | Yinchuan | 38.10 N 106.34 E |
| SAMN19699717 | NX09002 | 2009-03-28 | Pig | 0014520 (95%) | *Y.enterocolitica* | O: 3 | Yinchuan | 38.10 N 106.34 E |
| SAMN19699718 | NX09003 | 2009-03-28 | Pig | 0014520 (95%) | *Y.enterocolitica* | O: 3 | Yinchuan | 38.10 N 106.34 E |
| SAMN19699719 | NX09004 | 2009-03-28 | Pig | 0014520 (95%) | *Y.enterocolitica* | O: 3 | Yinchuan | 38.10 N 106.34 E |
| SAMN19699720 | NX09005 | 2009-03-28 | Pig | 0014520 (95%) | *Y.enterocolitica* | O: 3 | Yinchuan | 38.10 N 106.34 E |
| SAMN19699721 | NX09006 | 2009-03-28 | Pig | 0014520 (95%) | *Y.enterocolitica* | O: 3 | Yinchuan | 38.10 N 106.34 E |
| SAMN19699722 | NX09007 | 2009-04-22 | Pig | 1154723 (92.5%) | *Y.enterocolitica* | NA | Yinchuan | 38.10 N 106.34 E |
| SAMN19699723 | NX09008 | 2009-04-22 | Pig | 1154723 (92.5%) | *Y.enterocolitica* | O: 5 | Yinchuan | 38.10 N 106.34 E |
| SAMN19699724 | NX09009 | 2009-04-22 | Pig | 1154723 (92.5%) | *Y.enterocolitica* | O: 5 | Yinchuan | 38.10 N 106.34 E |
| SAMN19699725 | NX09010 | 2009-04-22 | Pig | 1154723 (92.5%) | *Y.enterocolitica* | O: 5 | Yinchuan | 38.10 N 106.34 E |
| SAMN19699726 | NX09011 | 2009-04-22 | Pig | 1154723 (92.5%) | *Y.enterocolitica* | O: 5 | Yinchuan | 38.10 N 106.34 E |
| SAMN19699727 | NX09013 | 2009-06-13 | Pig | 1154723 (92.5%) | *Y.enterocolitica* | O: 5 | Zhongwei | 36.56 N 105.64 E |
| SAMN19699728 | NX09014 | 2009-06-23 | Pig | 1154723 (92.5%) | *Y.enterocolitica* | O: 5 | Zhongwei | 36.56 N 105.64 E |
| SAMN19699729 | NX09017 | 2009-07-23 | Pig | 1154723 (92.5%) | *Y.enterocolitica* | O: 5 | Zhongwei | 36.56 N 105.64 E |
| SAMN19699730 | NX09018 | 2009-07-23 | Pig | 1154723 (92.5%) | *Y.enterocolitica* | O: 5 | Zhongwei | 36.56 N 105.64 E |
| SAMN19699731 | NX09019 | 2009-07-30 | Pig | 1154723 (92.5%) | *Y.intermedia* | NA | Zhongwei | 36.56 N 105.64 E |
| SAMN19699733 | NX09028 | 2009-08-15 | Pig | 0054523 (97.4%) | *Y.enterocolitica* | O: 9 | Zhongwei | 37.52 N 105.17 E |
| SAMN19699734 | NX09029 | 2009-08-15 | Pig | 0054522 (98.8%) | *Y.enterocolitica* | O: 9 | Zhongwei | 37.52 N 105.17 E |
| SAMN19699735 | NX09030 | 2009-08-15 | Pig | 0014522 (95%) | *Y.enterocolitica* | O: 3 | Zhongwei | 37.52 N 105.17 E |
| SAMN19699736 | NX09034 | 2009-08-23 | Pig | 1054122 (88.7%) | *Y.intermedia* | NA | Zhongwei | 37.52 N 105.17 E |
| SAMN19699737 | NX09035 | 2009-08-23 | Pig | 1054523 (81.1%) | *Y.massiliensis* | NA | Zhongwei | 37.52 N 105.17 E |
| BioSample | Samplename | Collection Date | Origin | Biochemical tests* | ANI analysis | Serotype | Location | [Coordinate](C:/Users/%E6%9C%88%E5%84%BF/AppData/Local/youdao/dict/Application/8.9.6.0/resultui/html/index.html#/javascript:;) |
| SAMN19699738 | NX09036 | 2009-08-09 | Pig | 0154723 (99.2%) | *Y.enterocolitica* | O: 5 | Zhongwei | 36.56 N 105.64 E |
| SAMN19699739 | NX09037 | 2009-08-09 | Pig | 0154723 (99.2%) | *Y.enterocolitica* | NA | Zhongwei | 36.56 N 105.64 E |
| SAMN19699740 | NX09038 | 2009-08-09 | Pig | 0154723 (99.2%) | *Y.enterocolitica* | O: 5 | Zhongwei | 36.56 N 105.64 E |
| SAMN19699741 | NX09039 | 2009-08-09 | Pig | 0154723 (99.2%) | *Y.enterocolitica* | NA | Zhongwei | 36.56 N 105.64 E |
| SAMN19699742 | NX09040 | 2009-08-09 | Pig | 0154723 (99.2%) | *Y.enterocolitica* | NA | Zhongwei | 36.56 N 105.64 E |
| SAMN19699743 | NX09041 | 2009-08-09 | Pig | 0154723 (99.2%) | *Y.enterocolitica* | NA | Zhongwei | 36.56 N 105.64 E |
| SAMN19699744 | NX09042 | 2009-08-10 | Pig | 0154723 (99.2%) | *Y.enterocolitica* | O: 5 | Zhongwei | 36.56 N 105.64 E |
| SAMN19699745 | NX09043 | 2009-08-10 | Pig | 0154723 (99.2%) | *Y.enterocolitica* | NA | Zhongwei | 36.56 N 105.64 E |
| SAMN19699746 | NX09044 | 2009-08-10 | Pig | 0154723 (99.2%) | *Y.enterocolitica* | NA | Zhongwei | 36.56 N 105.64 E |
| SAMN19699747 | NX09045 | 2009-08-10 | Pig | 0154723 (99.2%) | *Y.enterocolitica* | NA | Zhongwei | 36.56 N 105.64 E |
| SAMN19699748 | NX09047 | 2009-08-11 | Pig | 0154723 (99.2%) | *Y.enterocolitica* | NA | Zhongwei | 36.56 N 105.64 E |
| SAMN19699749 | NX09048 | 2009-08-12 | Pig | 1154723 (92.5%) | *Y.enterocolitica* | NA | Zhongwei | 36.56 N 105.64 E |
| SAMN19699750 | NX09057 | 2009-08-22 | Pig | 1154723 (92.5%) | *Y.enterocolitica* | NA | Zhongwei | 36.56 N 105.64 E |
| SAMN19699751 | NX09058 | 2009-08-22 | Pig | 1154723 (92.5%) | *Y.enterocolitica* | NA | Zhongwei | 36.56 N 105.64 E |
| SAMN19699752 | NX09059 | 2009-08-22 | Pig | 1154723 (92.5%) | *Y.enterocolitica* | NA | Zhongwei | 36.56 N 105.64 E |
| SAMN19699753 | NX09060 | 2009-08-23 | Pig | 1154723 (92.5%) | *Y.enterocolitica* | NA | Zhongwei | 36.56 N 105.64 E |
| SAMN19699754 | NX09061 | 2009-08-23 | Pig | 0154523 (98.9%) | *Y.enterocolitica* | NA | Zhongwei | 36.56 N 105.64 E |
| SAMN19699755 | NX09062 | 2009-08-23 | Pig | 0154723 (99.2%) | *Y.enterocolitica* | O: 8 | Zhongwei | 36.56 N 105.64 E |
| SAMN19699756 | NX10029 | 2010-08-26 | Pig | 0054723(98%) | *Y.massiliensis* | NA | Zhongwei | 36.56 N 105.64 E |
| SAMN19699757 | NX10034 | 2010-09-09 | Sheep | 0054723(98%) | *Y.massiliensis* | NA | Zhongwei | 36.56 N 105.64 E |
| SAMN19699758 | NX10039 | 2010-09-21 | Pig | 1054723 (81.5%) | *Y.massiliensis* | NA | Zhongwei | 37.52 N 105.17 E |
| SAMN19699759 | NX10040 | 2010-09-21 | Pig | 1054723 (81.5%) | *Y.massiliensis* | NA | Zhongwei | 37.52 N 105.17 E |
| SAMN19699760 | NX10041 | 2010-09-21 | Pig | 1054723 (81.5%) | *Y.proxima* | NA | Zhongwei | 37.52 N 105.17 E |
| SAMN19699761 | NX10042 | 2010-09-21 | Pig | 1054723 (81.5%) | *Y.massiliensis* | NA | Zhongwei | 37.52 N 105.17 E |
| SAMN19699762 | NX10043 | 2010-09-21 | Pig | 0014522 (95.0%) | *Y.enterocolitica* | NA | Zhongwei | 37.52 N 105.17 E |
| SAMN19699763 | NX11034 | 2011-07-15 | Pig | 1054723 (81.5%) | *Y.massiliensis* | NA | Zhongwei | 36.56 N 105.64 E |
| SAMN19699764 | NX11035 | 2011-07-15 | Pig | 1054723 (81.5%) | *Y.massiliensis* | NA | Zhongwei | 36.56 N 105.64 E |
| SAMN19699765 | NX11036 | 2011-07-15 | Pig | 1055723 (94.4%) | *Y.enterocolitica* | NA | Zhongwei | 36.56 N 105.64 E |
| SAMN19699766 | NX11037 | 2011-07-15 | Pig | 1054723 (81.5%) | *Y.massiliensis* | NA | Zhongwei | 36.56 N 105.64 E |
| SAMN19699767 | NX11038 | 2011-07-15 | Pig | 1054723 (81.5%) | *Y.massiliensis* | NA | Zhongwei | 36.56 N 105.64 E |
| SAMN19699768 | NX11039 | 2011-07-15 | Pig | 1054723 (81.5%) | *Y.massiliensis* | NA | Zhongwei | 36.56 N 105.64 E |
| SAMN19699770 | NX11049 | 2011-08-17 | Sheep | 1054723 (81.5%) | *Y.massiliensis* | NA | Zhongwei | 36.56 N 105.64 E |
| SAMN19699771 | NX11050 | 2011-08-17 | Sheep | 1254723 (34.8%) | *Y.massiliensis* | NA | Zhongwei | 36.56 N 105.64 E |
| SAMN19699772 | NX11051 | 2011-08-17 | Sheep | 1254723 (34.8%) | *Y.massiliensis* | NA | Zhongwei | 36.56 N 105.64 E |
| SAMN19699773 | NX11057 | 2011-10-19 | Food | 1155523 (98.3%) | *Y.intermedia* | Nag | Yinchuan | 38.47 N 106.27 E |
| SAMN19699774 | NX11058 | 2011-10-19 | Pig | 0014522 (95.0%) | *Y.enterocolitica* | O: 3 | Zhongwei | 36.56 N 105.64 E |
| SAMN19699775 | NX11070 | 2011-10-15 | Pig | 1014522 (93.9%) | *Y.enterocolitica* | O: 3 | Yinchuan | 38.47 N 106.27 E |
| SAMN19699776 | NX11071 | 2011-10-15 | Pig | 1014522 (93.9%) | *Y.enterocolitica* | O: 3 | Yinchuan | 38.47 N 106.27 E |
| SAMN19699777 | NX11072 | 2011-10-15 | Pig | 1014522 (93.9%) | *Y.enterocolitica* | O: 3 | Yinchuan | 38.47 N 106.27 E |
| SAMN19699778 | NX11073 | 2011-10-15 | Pig | 1014522 (93.9%) | *Y.enterocolitica* | O: 3 | Yinchuan | 38.47 N 106.27 E |
| SAMN19699779 | NX11074 | 2011-10-15 | Pig | 1014522 (93.9%) | *Y.enterocolitica* | O: 3 | Yinchuan | 38.47 N 106.27 E |
| SAMN19699780 | NX11075 | 2011-10-15 | Pig | 1014522 (93.9%) | *Y.enterocolitica* | O: 3 | Yinchuan | 38.47 N 106.27 E |
| BioSample | Samplename | Collection Date | Origin | Biochemical tests* | ANI analysis | Serotype | Location | [Coordinate](C:/Users/%E6%9C%88%E5%84%BF/AppData/Local/youdao/dict/Application/8.9.6.0/resultui/html/index.html#/javascript:;) |
| SAMN19699781 | NX11076 | 2011-10-15 | Pig | 1014522 (93.9%) | *Y.enterocolitica* | O: 3 | Yinchuan | 38.47 N 106.27 E |
| SAMN19699782 | NX11077 | 2011-10-15 | Pig | 1014522 (93.9%) | *Y.enterocolitica* | O: 3 | Yinchuan | 38.47 N 106.27 E |
| SAMN19699783 | NX11078 | 2011-10-15 | Pig | 1014522 (93.9%) | *Y.enterocolitica* | O: 3 | Yinchuan | 38.47 N 106.27 E |
| SAMN19699784 | NX11079 | 2011-10-15 | Pig | 1014522 (93.9%) | *Y.enterocolitica* | O: 3 | Yinchuan | 38.47 N 106.27 E |
| SAMN19699785 | NX11080 | 2011-10-15 | Pig | 1014522 (93.9%) | *Y.enterocolitica* | O: 3 | Yinchuan | 38.47 N 106.27 E |
| SAMN19699786 | NX11081 | 2011-10-15 | Pig | 1014522 (93.9%) | *Y.enterocolitica* | O: 3 | Yinchuan | 38.47 N 106.27 E |
| SAMN19699787 | NX11082 | 2011-10-15 | Pig | 1014522 (93.9%) | *Y.enterocolitica* | O: 3 | Yinchuan | 38.47 N 106.27 E |
| SAMN19699788 | NX11083 | 2011-10-15 | Pig | 1014522 (93.9%) | *Y.enterocolitica* | O: 3 | Yinchuan | 38.47 N 106.27 E |
| SAMN19699789 | NX11084 | 2011-10-15 | Pig | 1014522 (93.9%) | *Y.enterocolitica* | O: 3 | Yinchuan | 38.47 N 106.27 E |
| SAMN19699790 | NX11085 | 2011-10-15 | Pig | 1014522 (93.9%) | *Y.enterocolitica* | O: 3 | Yinchuan | 38.47 N 106.27 E |
| SAMN19699791 | NX11086 | 2011-10-15 | Pig | 1014522 (93.9%) | *Y.enterocolitica* | O: 3 | Yinchuan | 38.47 N 106.27 E |
| SAMN19699792 | NX11087 | 2011-10-15 | Pig | 1014522 (93.9%) | *Y.enterocolitica* | O: 3 | Yinchuan | 38.47 N 106.27 E |
| SAMN19699793 | NX11088 | 2011-10-15 | Pig | 1014522 (93.9%) | *Y.enterocolitica* | O: 3 | Yinchuan | 38.47 N 106.27 E |
| SAMN19699794 | NX11089 | 2011-10-15 | Pig | 1014522 (93.9%) | *Y.enterocolitica* | O: 3 | Yinchuan | 38.47 N 106.27 E |
| SAMN19699795 | NX11090 | 2011-10-15 | Pig | 1014522 (93.9%) | *Y.enterocolitica* | O: 3 | Yinchuan | 38.47 N 106.27 E |
| SAMN19699796 | NX11091 | 2011-10-15 | Pig | 1014522 (93.9%) | *Y.enterocolitica* | O: 3 | Yinchuan | 38.47 N 106.27 E |
| SAMN19699797 | NX11092 | 2011-10-15 | Pig | 1014522 (93.9%) | *Y.enterocolitica* | O: 3 | Yinchuan | 38.47 N 106.27 E |
| SAMN19699798 | NX11093 | 2011-10-15 | Pig | 1014522 (93.9%) | *Y.enterocolitica* | O: 3 | Yinchuan | 38.47 N 106.27 E |
| SAMN19699799 | NX11094 | 2011-10-15 | Pig | 1014522 (93.9%) | *Y.enterocolitica* | O: 3 | Yinchuan | 38.47 N 106.27 E |
| SAMN19699800 | NX12009 | 2012-07-12 | Cattle | 1054723 (92.5%) | *Y.enterocolitica* | NA | Zhongwei | 36.56 N 105.64 E |
| SAMN19699801 | NX12010 | 2012-07-12 | Cattle | 1054723 (92.5%) | *Y.enterocolitica* | NA | Zhongwei | 36.56 N 105.64 E |
| SAMN19699802 | NX12011 | 2012-07-12 | Cattle | 1054723 (92.5%) | *Y.enterocolitica* | NA | Zhongwei | 36.56 N 105.64 E |
| SAMN19699803 | NX12012 | 2012-07-12 | Chicken | 0154723 (99.2%) | *Y.enterocolitica* | O: 5 | Zhongwei | 36.56 N 105.64 E |
| SAMN19699804 | NX12013 | 2012-07-12 | Chicken | 0154723 (99.2%) | *Y.enterocolitica* | O: 5 | Zhongwei | 36.56 N 105.64 E |
| SAMN19699805 | NX12014 | 2012-07-12 | Sheep | 0154723 (99.2%) | *Y.enterocolitica* | NA | Zhongwei | 36.56 N 105.64 E |
| SAMN19699806 | NX12015 | 2012-07-12 | Sheep | 0154723 (99.2%) | *Y.enterocolitica* | NA | Zhongwei | 36.56 N 105.64 E |
| SAMN19699807 | NX12016 | 2012-07-12 | Food | 0354723 (93.1%) | *Y.enterocolitica* | O: 8 | Yinchuan | 38.47 N 106.27 E |
| SAMN19699808 | NX12017 | 2012-07-12 | Food | 0154723 (99.2%) | *Y.enterocolitica* | O: 9 | Yinchuan | 38.47 N 106.27 E |
| SAMN19699809 | NX12018 | 2012-07-12 | Food | 0154723 (99.2%) | *Y.enterocolitica* | NA | Yinchuan | 38.47 N 106.27 E |
| SAMN19699810 | NX12019 | 2012-07-12 | Food | 0154723 (99.2%) | *Y.enterocolitica* | NA | Yinchuan | 38.47 N 106.27 E |
| SAMN19699811 | NX12020 | 2012-07-12 | Sheep | 0154723 (99.2%) | *Y.enterocolitica* | NA | Zhongwei | 36.56 N 105.64 E |
| SAMN19699812 | NX12021 | 2012-07-12 | Sheep | 1054522 (97.9%) | *Y.intermedia* | NA | Zhongwei | 36.56 N 105.64 E |
| SAMN19699813 | NX12022 | 2012-07-12 | Sheep | 1054523 (81.1%) | *Y.intermedia* | NA | Zhongwei | 36.56 N 105.64 E |
| SAMN19699814 | NX12023 | 2012-07-12 | Cattle | 1054723 (92.5%) | *Y.enterocolitica* | NA | Zhongwei | 36.56 N 105.64 E |
| SAMN19699815 | NX12024 | 2012-07-12 | Cattle | 0154723 (99.2%) | *Y.enterocolitica* | O: 5 | Zhongwei | 36.56 N 105.64 E |
| SAMN19699816 | NX12025 | 2012-07-12 | Sheep | 0154723 (99.2%) | *Y.enterocolitica* | NA | Zhongwei | 36.56 N 105.64 E |
| SAMN19699817 | NX12036 | 2012-07-12 | Pig | 0054723 (98.0%) | *Y.enterocolitica* | O: 9 | Zhongwei | 36.56 N 105.64 E |
| SAMN19699818 | NX12056 | 2012-07-12 | Pig | 1014523 (96.4%) | *Y.enterocolitica* | O: 3 | Yinchuan | 38.47 N 106.27 E |
| SAMN19699819 | NX12057 | 2012-07-12 | Pig | 0014523 (97.7%) | *Y.enterocolitica* | O: 3 | Yinchuan | 38.47 N 106.27 E |
| SAMN19699820 | NX12058 | 2012-07-12 | Pig | 0014523 (95.0%) | *Y.enterocolitica* | NA | Yinchuan | 38.47 N 106.27 E |
| SAMN19699821 | NX12059 | 2012-07-12 | Pig | 0014523 (97.7%) | *Y.enterocolitica* | O: 3 | Yinchuan | 38.47 N 106.27 E |
| SAMN19699822 | NX12060 | 2012-07-12 | Pig | 0014523 (96.4%) | *Y.enterocolitica* | O: 3 | Yinchuan | 38.47 N 106.27 E |
| BioSample | Samplename | Collection Date | Origin | Biochemical tests* | ANI analysis | Serotype | Location | [Coordinate](C:/Users/%E6%9C%88%E5%84%BF/AppData/Local/youdao/dict/Application/8.9.6.0/resultui/html/index.html#/javascript:;) |
| SAMN19699823 | NX12061 | 2012-07-12 | Pig | 0014522 (95.0%) | *Y.enterocolitica* | O: 3 | Yinchuan | 38.47 N 106.27 E |
| SAMN19699824 | NX12062 | 2012-07-12 | Pig | 0014522 (95.0%) | *Y.enterocolitica* | O: 3 | Yinchuan | 38.47 N 106.27 E |
| SAMN19699825 | NX12063 | 2012-07-12 | Pig | 0014522 (95.0%) | *Y.enterocolitica* | O: 3 | Yinchuan | 38.47 N 106.27 E |
| SAMN19699826 | NX12064 | 2012-07-12 | Food | 0154723 (99.2%) | *Y.enterocolitica* | O: 8 | Yinchuan | 38.47 N 106.27 E |
| SAMN19699827 | NX12065 | 2012-07-12 | Food | 0154723 (99.2%) | *Y.enterocolitica* | O: 8 | Yinchuan | 38.47 N 106.27 E |
| SAMN19699828 | NX13049 | 2013-05-15 | Food | 0054723 (98.0%) | *Y.enterocolitica* | O: 5 | Yinchuan | 38.47 N 106.27 E |
| SAMN19699829 | NX13077 | 2013-08-29 | Pig | 0154723 (99.2%) | *Y.enterocolitica* | Nag | Zhongwei | 36.56 N 105.64 E |
| SAMN19699830 | NX14046 | 2014-06-10 | Rat | 0154723 (99.2%) | *Y.enterocolitica* | NA | Zhongwei | 36.56 N 105.64 E |
| SAMN19699831 | NX14047 | 2014-06-10 | Rat | 0054723 (98.0%) | *Y.enterocolitica* | NA | Zhongwei | 36.56 N 105.64 E |
| SAMN19699832 | NX14048 | 2014-06-10 | Rat | 1014523 (95.4%) | *Y.pekkanenii* | NA | Zhongwei | 36.56 N 105.64 E |
| SAMN19699833 | NX14049 | 2014-06-10 | Rat | 1015523 (93.8%) | *Y.pekkanenii* | NA | Zhongwei | 36.56 N 105.64 E |
| SAMN19699834 | NX14050 | 2014-06-10 | Rat | 0354723 (93.1%) | *Y.enterocolitica* | NA | Zhongwei | 36.56 N 105.64 E |
| SAMN19699835 | NX14051 | 2014-06-10 | Rat | 1114523 (99.7%) | *Y.pekkanenii* | NA | Zhongwei | 36.56 N 105.64 E |
| SAMN19699836 | NX14052 | 2014-06-10 | Rat | 1054723 (81.5%) | *Y.intermedia* | NA | Zhongwei | 36.56 N 105.64 E |
| SAMN19699837 | NX14053 | 2014-06-10 | Rat | 1054722 (98.6%) | *Y.intermedia* | O: 1, | Zhongwei | 36.56 N 105.64 E |
| SAMN19699838 | NX14054 | 2014-06-10 | Rat | 1114523 (99.7%) | *Y.pekkanenii* | NA | Zhongwei | 36.56 N 105.64 E |
| SAMN19699839 | NX14055 | 2014-06-10 | Rat | 1054723 (81.5%) | *Y.intermedia* | O: 1,2 | Zhongwei | 36.56 N 105.64 E |
| SAMN19699840 | NX14056 | 2014-06-10 | Rat | 0154623 (99.2%) | *Y.enterocolitica* | NA | Zhongwei | 36.56 N 105.64 E |
| SAMN19699842 | NX14083 | 2014-07-24 | Rat | 1054723 (81.5%) | *Y.massiliensis* | NA | Zhongwei | 36.56 N 105.64 E |
| SAMN19699843 | NX14084 | 2014-07-24 | Rat | 1114523 (99.7%) | *Y.pekkanenii* | O: 1,2 | Zhongwei | 36.56 N 105.64 E |
| SAMN19699844 | NX14085 | 2014-07-24 | Rat | 1054723 (81.5%) | *Y.massiliensis* | NA | Zhongwei | 36.56 N 105.64 E |
| SAMN19699847 | NX14109 | 2014-09-29 | Sheep | 0014523 (97.7%) | *Y.enterocolitica* | O: 9 | Zhongwei | 36.56 N 105.64 E |
| SAMN19699848 | NX14111 | 2014-09-29 | Sheep | 0014523 (97.7%) | *Y.enterocolitica* | O: 9 | Zhongwei | 36.56 N 105.64 E |
| SAMN19699849 | NX14112 | 2014-09-29 | Sheep | 0014723 (98.6%) | *Y.enterocolitica* | O: 5 | Zhongwei | 36.56 N 105.64 E |
| SAMN19699850 | NX14113 | 2014-09-29 | Pig | 0154723 (99.2%) | *Y.enterocolitica* | O: 5 | Zhongwei | 36.56 N 105.64 E |
| SAMN19699851 | NX14114 | 2014-09-29 | Pig | 0154723 (99.2%) | *Y.enterocolitica* | NA | Zhongwei | 36.56 N 105.64 E |
| SAMN19699852 | NX14115 | 2014-09-29 | Sheep | 0154723 (99.2%) | *Y.enterocolitica* | O: 8 | Zhongwei | 36.56 N 105.64 E |
| SAMN19699853 | NX14116 | 2014-09-29 | Sheep | 0154723 (99.2%) | *Y.enterocolitica* | O: 8 | Zhongwei | 36.56 N 105.64 E |
| SAMN19699854 | NX14117 | 2014-09-29 | Sheep | 0154723 (99.2%) | *Y.enterocolitica* | O: 8 | Zhongwei | 36.56 N 105.64 E |
| SAMN19699855 | NX14118 | 2014-09-29 | Sheep | 0154723 (99.2%) | *Y.enterocolitica* | O: 8 | Zhongwei | 36.56 N 105.64 E |
| SAMN19699856 | NX14119 | 2014-09-29 | Pig | 1054723 (81.5%) | *Y.mollaretii* | NA | Zhongwei | 36.56 N 105.64 E |
| SAMN19699857 | NX14120 | 2014-09-29 | Pig | 1054723 (81.5%) | *Y.massiliensis* | O: 9 | Zhongwei | 36.56 N 105.64 E |
| SAMN19699858 | NX14121 | 2014-09-29 | Pig | 0154723 (99.2%) | *Y.enterocolitica* | NA | Zhongwei | 36.56 N 105.64 E |
| SAMN19699859 | NX15095 | 2015-08-13 | Sheep | 0154723 (99.2%) | *Y.enterocolitica* | O: 5 | Zhongwei | 36.56 N 105.64 E |
| SAMN19699860 | NX15096 | 2015-08-13 | Sheep | 0154723 (99.2%) | *Y.enterocolitica* | O: 8 | Zhongwei | 36.56 N 105.64 E |
| SAMN19699861 | NX15097 | 2015-08-13 | Sheep | 0154723 (99.2%) | *Y.enterocolitica* | O: 8 | Zhongwei | 36.56 N 105.64 E |
| SAMN19699862 | NX15098 | 2015-08-13 | Sheep | 0154723 (99.2%) | *Y.enterocolitica* | O: 8 | Zhongwei | 36.56 N 105.64 E |
| SAMN19699863 | NX15099 | 2015-08-13 | Sheep | 0154723 (99.2%) | *Y.enterocolitica* | O:5,8,9 | Zhongwei | 36.56 N 105.64 E |
| SAMN19699864 | NX15100 | 2015-08-13 | Sheep | 0154723 (99.2%) | *Y.enterocolitica* | O: 8 | Zhongwei | 36.56 N 105.64 E |
| SAMN19699865 | NX15101 | 2015-08-13 | Sheep | 0154723 (99.2%) | *Y.mollaretii* | Nag | Zhongwei | 36.56 N 105.64 E |
| SAMN19699866 | NX15105 | 2015-08-27 | Sheep | 0154723 (99.2%) | *Y.mollaretii* | NA | Zhongwei | 36.56 N 105.64 E |
| BioSample | Samplename | Collection Date | Origin | Biochemical tests* | ANI analysis | Serotype | Location | [Coordinate](C:/Users/%E6%9C%88%E5%84%BF/AppData/Local/youdao/dict/Application/8.9.6.0/resultui/html/index.html#/javascript:;) |
| SAMN19699867 | NX15106 | 2015-08-27 | Sheep | 0154723 (95.0%) | *Y.proxima* | NA | Zhongwei | 36.56 N 105.64 E |
| SAMN19699868 | NX15107 | 2015-09-15 | Sheep | 0154723 (99.2%) | *Y.rochesterensis* | NA | Zhongwei | 36.56 N 105.64 E |
| SAMN19699869 | NX15108 | 2015-08-27 | Rat | 0154723 (99.2%) | *Y.enterocolitica* | NA | Zhongwei | 36.56 N 105.64 E |
| SAMN19699870 | NX15131 | 2015-09-15 | Pig | 1054723 (81.5%) | *Y.massiliensis* | NA | Zhongwei | 36.56 N 105.64 E |
| SAMN19699871 | NX15132 | 2015-09-15 | Pig | 0155723 (99.8%) | *Y.enterocolitica* | NA | Zhongwei | 36.56 N 105.64 E |
| SAMN19699872 | NX15133 | 2015-09-15 | Pig | 1054723 (81.5%) | *Y.massiliensis* | NA | Zhongwei | 36.56 N 105.64 E |
| SAMN19699873 | NX 16034 | 2016-08-18 | Food | 0014503 (92.7%) | *Y.enterocolitica* | NA | Yinchuan | 38.47 N 106.27 E |
| SAMN19699874 | NX16044 | 2016-08-26 | Cattle | 0154723 (99.2%) | *Y.enterocolitica* | O: 5 | Zhongwei | 36.56 N 105.64 E |
| SAMN19699875 | NX16081 | 2016-10-13 | Pig | 0014522 (95%) | *Y.enterocolitica* | NA | Zhongwei | 36.56 N 105.64 E |
| SAMN19699876 | NX16082 | 2016-10-13 | Pig | 0014522 (95%) | *Y.enterocolitica* | NA | Zhongwei | 36.56 N 105.64 E |
| SAMN19699877 | NX16083 | 2016-10-13 | Pig | 0014522 (95%) | *Y.enterocolitica* | NA | Zhongwei | 36.56 N 105.64 E |
| SAMN19699878 | NX16084 | 2016-10-13 | Pig | 0014520 (87.6%) | *Y.enterocolitica* | NA | Zhongwei | 36.56 N 105.64 E |
| SAMN19699879 | NX16085 | 2016-10-13 | Pig | 0054723 (98%) | *Y.massiliensis* | NA | Zhongwei | 36.56 N 105.64 E |
| SAMN19699880 | NX16106 | 2016-12-18 | Food | 1354723 (92.7%) | *Y.enterocolitica* | NA | Yinchuan | 38.47 N 106.27 E |
| SAMN19699881 | NX17015 | 2017-05-23 | Food | 1155723 (98.3%) | *Y.intermedia* | NA | Yinchuan | 38.47 N 106.27 E |
| SAMN19699882 | NX17016 | 2017-05-23 | Food | 1155723 (98.3%) | *Y.intermedia* | NA | Yinchuan | 38.47 N 106.27 E |
| SAMN19699883 | NX17017 | 2017-05-23 | Food | 1155723 (98.3%) | *Y.intermedia* | NA | Yinchuan | 38.47 N 106.27 E |
| SAMN19699884 | NX17027 | 2017-07-12 | Chicken | 0154723 (99.2%) | *Y.enterocolitica* | NA | Zhongwei | 36.56 N 105.64 E |
| SAMN19699885 | NX17061 | 2017-10-17 | Pig | 0114723 (99.5%) | *Y.enterocolitica* | O: 5 | Zhongwei | 36.56 N 105.64 E |
| SAMN19699886 | NX17062 | 2017-10-17 | Pig | 1115723 (97.9%) | *Y.enterocolitica* | O: 5 | Zhongwei | 36.56 N 105.64 E |
| SAMN19699887 | NX17063 | 2017-10-17 | Pig | 0114523 (99.9%) | *Y.enterocolitica* | O: 5 | Zhongwei | 36.56 N 105.64 E |
| SAMN19699888 | NX17064 | 2017-10-17 | Pig | 0114523 (99.9%) | *Y.enterocolitica* | O: 5 | Zhongwei | 36.56 N 105.64 E |
| SAMN19699889 | NX17065 | 2017-10-17 | Pig | 0014523 (97.7%) | *Y.mollaretii* | NA | Zhongwei | 36.56 N 105.64 E |
| SAMN19699890 | NX17066 | 2017-10-17 | Pig | 0014523 (97.7%) | *Y.canariae* | NA | Zhongwei | 36.56 N 105.64 E |
| SAMN19699891 | NX17067 | 2017-10-17 | Pig | 0115723 (99.5%) | *Y.enterocolitica* | O: 5 | Zhongwei | 36.56 N 105.64 E |
| SAMN19699892 | NX17068 | 2017-10-17 | Pig | 1014723 (98.7%) | *Y.massiliensis* | NA | Zhongwei | 36.56 N 105.64 E |
| SAMN19699893 | NX17069 | 2017-10-17 | Pig | 1014723 (98.7%) | *Y.massiliensis* | NA | Zhongwei | 36.56 N 105.64 E |
| SAMN19699894 | NX17070 | 2017-10-17 | Pig | 1054523 (81.1%) | *Y.massiliensis* | NA | Zhongwei | 36.56 N 105.64 E |
| SAMN19699895 | NX17071 | 2017-10-17 | Pig | 0154723 (99.7%) | *Y.enterocolitica* | O: 5 | Zhongwei | 36.56 N 105.64 E |
| SAMN19699896 | NX17072 | 2017-10-17 | Pig | 1054523 (81.1%) | *Y.massiliensis* | O: 1,2 | Zhongwei | 36.56 N 105.64 E |
| SAMN19699897 | NX17073 | 2017-10-17 | Pig | 1054523 (81.1%) | *Y.massiliensis* | NA | Zhongwei | 36.56 N 105.64 E |
| SAMN19699898 | NX17074 | 2017-10-17 | Pig | 0054723 (99.8%) | *Y.enterocolitica* | NA | Zhongwei | 36.56 N 105.64 E |
| SAMN19699899 | NX17075 | 2017-10-17 | Pig | 0114723 (99.9%) | *Y.mollaretii* | NA | Zhongwei | 36.56 N 105.64 E |
| SAMN19699900 | NX17076 | 2017-10-17 | Pig | 1054723 (81.5%) | *Y.massiliensis* | NA | Zhongwei | 36.56 N 105.64 E |
| SAMN19699901 | NX18077 | 2018-05-17 | Food | 0154723 (99.2%) | *Y.enterocolitica* | O: 53 | Yinchuan | 38.47 N 106.27 E |
| SAMN19699902 | NX18078 | 2018-05-17 | Food | 0154723 (81.5%) | *Y.massiliensis* | Nag | Yinchuan | 38.47 N 106.27 E |
| SAMN19699903 | NX18079 | 2018-05-17 | Food | 0154723 (81.5%) | *Y.massiliensis* | Nag | Yinchuan | 38.47 N 106.27 E |
| SAMN19699904 | NX18080 | 2018-05-17 | Food | 0154723 (99.2%) | *Y.enterocolitica* | Nag | Yinchuan | 38.47 N 106.27 E |
| SAMN19699905 | NX18081 | 2018-05-17 | Food | 0154723 (81.5%) | *Y.intermedia* | NA | Yinchuan | 38.47 N 106.27 E |
| SAMN19699906 | NX18083 | 2018-06-07 | Human | 0154723 (99.8%) | *Y.proxima* | Nag | Wuzhong | 38.02 N 106.08 E |
| SAMN19699907 | NX18084 | 2018-06-12 | Human | 0014522 (95%) | *Y.enterocolitica* | Nag | Shizuishan | 39.02 N 106.37 E |
| BioSample | Samplename | Collection Date | Origin | Biochemical tests* | ANI analysis | Serotype | Location | [Coordinate](C:/Users/%E6%9C%88%E5%84%BF/AppData/Local/youdao/dict/Application/8.9.6.0/resultui/html/index.html#/javascript:;) |
| SAMN19699908 | NX18085 | 2018-06-12 | Human | 0014522 (95%) | *Y.enterocolitica* | Nag | Shizuishan | 39.02 N 106.37 E |
| SAMN19699909 | NX18106 | 2018-07-10 | Human | 0154723 (99.2%) | *Y.enterocolitica* | O: 1,2,5 | Guyuan | 36.00 N 106.29 E |
| SAMN19699910 | NX18107 | 2018-07-10 | Human | 0154723 (95%) | *Y.enterocolitica* | O: 9 | Guyuan | 36.00 N 106.29 E |
| SAMN19699911 | NX18108 | 2018-07-10 | Human | 0014522 (95.0%) | *Y.enterocolitica* | NA | Zhongwei | 37.52 N 105.17 E |
| SAMN19699912 | NX18109 | 2018-07-10 | Human | 0014522 (95.0%) | *Y.enterocolitica* | NA | Shizuishan | 39.02 N 106.37 E |
| SAMN19699913 | NX18110 | 2018-07-10 | Human | 0014522 (95.0%) | *Y.enterocolitica* | NA | Shizuishan | 39.02 N 106.37 E |
| SAMN19699914 | NX18118 | 2018-08-07 | Human | 0014522 (95.0%) | *Y.enterocolitica* | Na | Shizuishan | 39.02 N 106.37 E |
| SAMN19699915 | NX18126 | 2018-08-30 | Food | 1054723 (81.5%) | *Y.intermedia* | NA | Yinchuan | 38.47 N 106.27 E |
| SAMN19699916 | NX18127 | 2018-08-30 | Sheep | 1054723 (81.5%) | *Y.intermedia* | NA | Zhongwei | 36.56 N 105.64 E |
| SAMN19699917 | NX18128 | 2018-08-30 | Sheep | 1054523 (81.1%) | *Y.intermedia* | NA | Zhongwei | 36.56 N 105.64 E |
| SAMN19699918 | NX18129 | 2018-08-30 | Sheep | 1055723 (94.4%) | *Y.intermedia* | NA | Zhongwei | 36.56 N 105.64 E |
| SAMN19699919 | NX18130 | 2018-08-30 | Sheep | 1054723 (81.5%) | *Y.intermedia* | NA | Zhongwei | 36.56 N 105.64 E |
| SAMN19699920 | NX18131 | 2018-08-30 | Sheep | 1054523 (81.1%) | *Y.massiliensis* | NA | Zhongwei | 36.56 N 105.64 E |
| SAMN19699921 | NX18160 | 2018-10-16 | Pig | 1014522 (93.9%) | *Y.enterocolitica* | O: 3 | Zhongwei | 36.56 N 105.64 E |
| SAMN19699922 | NX18161 | 2018-10-16 | Pig | 1014522 (93.9%) | *Y.enterocolitica* | O: 3 | Zhongwei | 36.56 N 105.64 E |
| SAMN19699923 | NX18162 | 2018-10-16 | Pig | 1014522 (93.9%) | *Y.enterocolitica* | O: 3 | Zhongwei | 36.56 N 105.64 E |
| SAMN19699924 | NX18163 | 2018-10-16 | Pig | 1014522 (93.9%) | *Y.enterocolitica* | O: 3 | Zhongwei | 36.56 N 105.64 E |
| SAMN19699925 | NX18164 | 2018-10-16 | Pig | 1014522 (93.9%) | *Y.enterocolitica* | O: 3 | Zhongwei | 36.56 N 105.64 E |
| SAMN19699926 | NX18165 | 2018-10-16 | Pig | 1014522 (93.9%) | *Y.enterocolitica* | O: 3 | Zhongwei | 36.56 N 105.64 E |
| SAMN19699927 | NX18166 | 2018-10-16 | Pig | 1014522 (93.9%) | *Y.enterocolitica* | O: 3 | Zhongwei | 36.56 N 105.64 E |
| SAMN19699928 | NX18167 | 2018-10-16 | Pig | 1014522 (93.9%) | *Y.enterocolitica* | O: 3 | Zhongwei | 36.56 N 105.64 E |
| SAMN19699929 | NX18168 | 2018-10-16 | Pig | 1014522 (93.9%) | *Y.enterocolitica* | O: 3 | Zhongwei | 36.56 N 105.64 E |
| SAMN19699930 | NX18169 | 2018-10-16 | Pig | 1014522 (93.9%) | *Y.enterocolitica* | O: 3 | Zhongwei | 36.56 N 105.64 E |
| SAMN19699931 | NX18170 | 2018-10-16 | Pig | 0014522 (95.0%) | *Y.enterocolitica* | O: 3 | Zhongwei | 36.56 N 105.64 E |
| SAMN19699932 | NX18171 | 2018-10-16 | Pig | 1014522 (93.1%) | *Y.enterocolitica* | O: 3 | Zhongwei | 36.56 N 105.64 E |
| SAMN19699933 | NX18172 | 2018-10-16 | Pig | 1014522 (93.1%) | *Y.enterocolitica* | O: 3 | Zhongwei | 36.56 N 105.64 E |
| SAMN19699934 | NX18173 | 2018-10-16 | Pig | 1054723 (81.5%) | *Y.massiliensis* | NA | Zhongwei | 36.56 N 105.64 E |
| SAMN19699935 | NX18174 | 2018-10-16 | Hamster | 1014522 (93.1%) | *Y.mollaretii* | NA | Zhongwei | 36.56 N 105.64 E |
| SAMN19699936 | NX18175 | 2018-10-16 | Hamster | 1014522 (93.2%) | *Y.mollaretii* | NA | Zhongwei | 36.56 N 105.64 E |
| SAMN19699937 | NX18176 | 2018-10-16 | Human | 0014522 (95.0%) | *Y.enterocolitica* | O: 3 | Guyuan | 35.85 N 106.64 E |
| SAMN19699938 | NX18189 | 2018-12-27 | Food | 1055523 (95.4%) | *Y.intermedia* | NA | Yinchuan | 38.47 N 106.27 E |
| SAMN19699939 | NX18190 | 2018-12-27 | Food | 1055723 (94.4%) | *Y.intermedia* | NA | Yinchuan | 38.47 N 106.27 E |
| SAMN19699940 | NX19005 | 2019-03-14 | Food | 0154723 (99.2%) | *Y.enterocolitica* | Nag | Yinchuan | 38.47 N 106.27 E |
| SAMN19699941 | NX19006 | 2019-03-14 | Food | 0154723 (99.3%) | *Y.enterocolitica* | Nag | Yinchuan | 38.47 N 106.27 E |
| SAMN19699942 | NX19017 | 2019-03-20 | Food | 1054723 (81.5%) | *Y.frederiksenii* | Nag | Shizuishan | 39.02 N 106.37 E |
| SAMN19699943 | NX19018 | 2019-03-25 | Food | 0154723 (99.0%) | *Y.enterocolitica* | O: 5 | Shizuishan | 39.02 N 106.37 E |
| SAMN19699944 | NX19019 | 2019-03-29 | Human | 0014522 (95.0%) | *Y.enterocolitica* | Nag | Yinchuan | 38.47 N 106.27 E |
| SAMN19699945 | NX19027 | 2019-06-25 | Food | 1166723 (98.3%) | *Y.intermedia* | NA | Yinchuan | 38.47 N 106.27 E |
| SAMN19699946 | NX19028 | 2019-06-25 | Food | 1166723 (98.3%) | *Y.intermedia* | NA | Yinchuan | 38.47 N 106.27 E |
| SAMN19699947 | NX19029 | 2019-06-25 | Food | 0154723 (99.2%) | *Y.enterocolitica* | NA | Yinchuan | 38.47 N 106.27 E |
| SAMN19699948 | NX19042 | 2019-07-10 | Food | 1154723 (92.5%) | *Y.enterocolitica* | NA | Yinchuan | 38.47 N 106.27 E |
| SAMN19699949 | NX19043 | 2019-07-10 | Food | 1154723 (92.5%) | *Y.enterocolitica* | NA | Yinchuan | 38.47 N 106.27 E |
| BioSample | Samplename | Collection Date | Origin | Biochemical tests* | ANI analysis | Serotype | Location | [Coordinate](C:/Users/%E6%9C%88%E5%84%BF/AppData/Local/youdao/dict/Application/8.9.6.0/resultui/html/index.html#/javascript:;) |
| SAMN19699950 | NX19044 | 2019-07-10 | Food | 1054723 (81.5%) | *Y.kristensenii* | NA | Yinchuan | 38.47 N 106.27 E |
| SAMN19699951 | NX19045 | 2019-07-10 | Food | 1154723 (92.5%) | *Y.enterocolitica* | NA | Yinchuan | 38.47 N 106.27 E |
| SAMN19699952 | NX19046 | 2019-07-10 | Food | 1055723 (94.4%) | *Y.intermedia* | NA | Yinchuan | 38.47 N 106.27 E |
| SAMN19699953 | NX19047 | 2019-07-10 | Food | 1055723 (94.4%) | *Y.intermedia* | NA | Yinchuan | 38.47 N 106.27 E |
| SAMN19699954 | NX19048 | 2019-07-10 | Food | 1154723 (92.5%) | *Y.enterocolitica* | NA | Yinchuan | 38.47 N 106.27 E |
| SAMN19699955 | NX19049 | 2019-07-19 | Food | 1154723 (92.5%) | *Y. alsatica* | NA | Yinchuan | 38.47 N 106.27 E |
| SAMN19699956 | NX19050 | 2019-07-19 | Food | 1154723 (92.5%) | *Y.intermedia* | NA | Yinchuan | 38.47 N 106.27 E |
| SAMN19699957 | NX19051 | 2019-07-19 | Food | 1154723 (92.5%) | *Y.intermedia* | NA | Yinchuan | 38.47 N 106.27 E |
| SAMN19699958 | NX19052 | 2019-07-19 | Food | 1154723 (92.5%) | *Y.intermedia* | NA | Yinchuan | 38.47 N 106.27 E |
| SAMN19699959 | NX19053 | 2019-07-19 | Food | 1154723 (92.5%) | *Y.enterocolitica* | NA | Yinchuan | 38.47 N 106.27 E |
| SAMN19699960 | NX19054 | 2019-07-19 | Food | 1154723 (92.5%) | *Y.proxima* | NA | Yinchuan | 38.47 N 106.27 E |
| SAMN19699961 | NX19055 | 2019-07-19 | Food | 1154723 (92.5%) | *Y.intermedia* | NA | Yinchuan | 38.47 N 106.27 E |
| SAMN19699962 | NX19056 | 2019-07-19 | Food | 1154723 (92.5%) | *Y. alsatica* | NA | Yinchuan | 38.47 N 106.27 E |
| SAMN19699963 | NX19057 | 2019-07-19 | Food | 1055523 (95.4%) | *Y.intermedia* | NA | Yinchuan | 38.47 N 106.27 E |
| SAMN19699966 | NX19106 | 2019-12-27 | Human | 0154723 (99.2%) | *Y.enterocolitica* | Nag | Zhongwei | 36.56 N 105.64 E |
| SAMN19699967 | NX19107 | 2019-12-27 | Human | 0154722 (99.2%) | *Y.enterocolitica* | O: 8 | Zhongwei | 36.56 N 105.64 E |
| SAMN19699968 | NX19108 | 2019-09-20 | Food | 0154723 (99.2%) | *Y.intermedia* | O: 5 | Yinchuan | 38.47 N 106.27 E |
| SAMN19699969 | NX19109 | 2019-09-20 | Food | 0154723 (99.2%) | *Y.enterocolitica* | O: 8 | Yinchuan | 38.47 N 106.27 E |
| SAMN19699970 | NX19110 | 2019-09-20 | Food | 0154723 (99.2%) | *Y.intermedia* | Nag | Yinchuan | 38.47 N 106.27 E |
| SAMN19699971 | NX19111 | 2019-10-15 | Food | 0154723 (99.2%) | *Y.enterocolitica* | O: 9 | Yinchuan | 38.47 N 106.27 E |
| SAMN19699972 | NX19112 | 2019-10-15 | Food | 0154723 (99.2%) | *Y.enterocolitica* | O: 5 | Yinchuan | 38.47 N 106.27 E |

Note: NA, not applicable; Nag, nonagglutinative. Biochemical tests*，biochemical spectrum and identification percentages.

**Table S6** Origin, serotype, STs, and CTs of 187 strains of *Y. enterocolitica*

| ST | CT | Samplename | Origin | Biotype | Serotype |
| --- | --- | --- | --- | --- | --- |
| 429 | 4609 | NX18160 | Pig | 4 | O: 3 |
|  | 4609 | NX18161 | Pig | 4 | O: 3 |
|  | 4609 | NX18163 | Pig | 4 | O: 3 |
|  | 4609 | NX18164 | Pig | 4 | O: 3 |
|  | 4609 | NX18165 | Pig | 4 | O: 3 |
|  | 4609 | NX18167 | Pig | 4 | O: 3 |
|  | 4609 | NX18168 | Pig | 4 | O: 3 |
|  | 4609 | NX18169 | Pig | 4 | O: 3 |
|  | 4609 | NX18171 | Pig | 4 | O: 3 |
|  | 4609 | NX18166 | Pig | 4 | O: 3 |
|  | 4506 | NX09001 | Pig | 4 | O: 3 |
|  | 4506 | NX09002 | Pig | 4 | O: 3 |
|  | 4506 | NX09003 | Pig | 4 | O: 3 |
|  | 4506 | NX09004 | Pig | 4 | O: 3 |
|  | 4506 | NX09005 | Pig | 4 | O: 3 |
|  | 4506 | NX09006 | Pig | 4 | O: 3 |
|  | 4493 | NX0740 | Pig | 4 | O: 3 |
|  | 4493 | NX0742 | Pig | 4 | O: 3 |
|  | 4493 | NX0746 | Pig | 4 | O: 3 |
|  | 4493 | NX0750 | Pig | 4 | O: 3 |
|  | 4493 | NX0752 | Pig | 3 | O: 3 |
|  | 4534 | NX11071 | Pig | 4 | O: 3 |
|  | 4534 | NX11072 | Pig | 4 | O: 3 |
|  | 4534 | NX11074 | Pig | 4 | O: 3 |
|  | 4534 | NX11078 | Pig | 4 | O: 3 |
|  | 4534 | NX11082 | Pig | 3 | O: 3 |
|  | 4536 | NX11073 | Pig | 4 | O: 3 |
|  | 4536 | NX11077 | Pig | 4 | O: 3 |
|  | 4536 | NX11081 | Pig | 4 | O: 3 |
|  | 4536 | NX11083 | Pig | 4 | O: 3 |
|  | 4536 | NX11091 | Pig | 4 | O: 3 |
|  | 4561 | NX12058 | Pig | 4 | O: 3 |
|  | 4561 | NX12060 | Pig | 4 | O: 3 |
|  | 4561 | NX12061 | Pig | 4 | O: 3 |
|  | 4561 | NX12062 | Pig | 4 | O: 3 |
|  | 4589 | NX16081 | Pig | 4 | O: 3 |
|  | 4589 | NX16082 | Pig | 4 | O: 3 |
|  | 4589 | NX16083 | Pig | 4 | O: 3 |
|  | 4589 | NX16084 | Pig | 4 | O: 3 |
|  | 4497 | NX0745 | Pig | 4 | O: 3 |
|  | 4497 | NX0749 | Pig | 4 | O: 3 |
|  | 4497 | NX0751 | Pig | 4 | O: 3 |
| ST | CT | Samplename | Origin | Biotype | Serotype |
| 429 | 4497 | NX0747 | Pig | 4 | O: 3 |
|  | 4537 | NX11075 | Pig | 4 | O: 3 |
|  | 4537 | NX11080 | Pig | 4 | O: 3 |
|  | 4537 | NX11085 | Pig | 4 | O: 3 |
|  | 4543 | NX11089 | Pig | 3 | O: 3 |
|  | 4543 | NX11090 | Pig | 4 | O: 3 |
|  | 4543 | NX11093 | Pig | 4 | O: 3 |
|  | 4502 | NX0753 | Pig | 4 | O: 3 |
|  | 4502 | NX0756 | Pig | 4 | O: 3 |
|  | 4539 | NX11079 | Pig | 4 | O: 3 |
|  | 4539 | NX11087 | Pig | 4 | O: 3 |
|  | 4544 | NX11092 | Pig | 4 | O: 3 |
|  | 4544 | NX11094 | Pig | 4 | O: 3 |
|  | 4560 | NX12057 | Pig | 4 | O: 3 |
|  | 4560 | NX12059 | Pig | 4 | O: 3 |
|  | 4603 | NX18084 | Human | 4 | Nag |
|  | 4603 | NX18109 | Human | 4 | O: 3 |
|  | 4492 | NX0738 | Pig | 4 | O: 3 |
|  | 4494 | NX0741 | Pig | 4 | O: 3 |
|  | 4495 | NX0743 | Pig | 4 | O: 3 |
|  | 4496 | NX0744 | Pig | 4 | O: 3 |
|  | 4498 | NX0748 | Pig | 4 | O: 3 |
|  | 4503 | NX0754 | Pig | 4 | O: 3 |
|  | 4505 | NX08020 | Pig | 4 | O: 3 |
|  | 4516 | NX09030 | Pig | 4 | O: 3 |
|  | 4533 | NX11070 | Pig | 4 | O: 3 |
|  | 4538 | NX11076 | Pig | 4 | O: 3 |
|  | 4540 | NX11084 | Pig | 4 | O: 3 |
|  | 4541 | NX11086 | Pig | 4 | O: 3 |
|  | 4542 | NX11088 | Pig | 4 | O: 3 |
|  | 4559 | NX12056 | Pig | 3 | O: 3 |
|  | 4562 | NX12063 | Pig | 4 | O: 3 |
|  | 4604 | NX18085 | Human | 4 | Nag |
|  | 4607 | NX18108 | Human | 4 | O: 3 |
|  | 4608 | NX18110 | Human | 4 | O: 3 |
|  | 4610 | NX18118 | Human | 4 | O: 3 |
|  | 4611 | NX18162 | Pig | 4 | O: 3 |
|  | 4614 | NX18170 | Pig | 4 | O: 3 |
|  | 4615 | NX18172 | Pig | 4 | O: 3 |
|  | 4616 | NX18176 | Human | 4 | O: 3 |
|  | 4746 | NX10043 | Pig | 4 | O: 3 |
| 3 | 4509 | NX09009 | Pig | 1A | O: 5 |
|  | 4509 | NX09010 | Pig | 1A | O: 5 |
| ST | CT | Samplename | Origin | Biotype | Serotype |
| 3 | 4509 | NX09011 | Pig | 1A | O: 5 |
|  | 4750 | NX12017 | Food | 1A | O: 9 |
|  | 4579 | NX15095 | Sheep | 1A | O: 5 |
|  | 4594 | NX17062 | Pig | 1A | O: 5 |
|  | 4598 | NX17071 | Pig | 1A | O: 5 |
|  | 4618 | NX19018 | Food | 1A | O: 5 |
|  | 4759 | NX19029 | Food | 1A | O: 5 |
| 13 | 4570 | NX14109 | Sheep | 5 | O: 9 |
|  | 4570 | NX14111 | Sheep | 5 | O: 9 |
|  | 4576 | NX14117 | Sheep | 5 | O: 8 |
|  | 4576 | NX14118 | Sheep | 5 | O: 8 |
|  | 4571 | NX14112 | Sheep | 5 | O: 5 |
|  | 4752 | NX14115 | Sheep | 5 | O: 8 |
|  | 4575 | NX14116 | Sheep | 5 | O: 8 |
| 278 | 4527 | NX09047 | Pig | 1A | O: 5 |
|  | 4585 | NX15108 | Rat | 1A | O: 5 |
|  | 4754 | NX16034 | Food | 1A | O: 5 |
|  | 4755 | NX17061 | Pig | 1A | O: 5 |
|  | 4599 | NX17074 | Pig | 1A | O: 5 |
|  | 4758 | NX19006 | Food | 1A | Nag |
| 178 | 4630 | NX19109 | Food | 1A | O: 8 |
|  | 4630 | NX19111 | Food | 1A | O: 9 |
|  | 4744 | NX09062 | Pig | 1A | O: 8 |
|  | 4551 | NX12016 | Food | 1A | O: 8 |
|  | 4591 | NX16106 | Food | 1A | O: 8 |
| 637 | 4550 | NX12014 | Sheep | 1A | O: 8 |
|  | 4550 | NX12015 | Sheep | 1A | O: 8 |
|  | 4550 | NX12020 | Sheep | 1A | O: 8 |
|  | 4550 | NX12025 | Sheep | 1A | O: 8 |
|  | 4592 | NX17027 | Chicken | 1A | O: 8 |
| 640 | 4568 | NX14047 | Rat | 1A | O: 5 |
|  | 4568 | NX14050 | Rat | 1A | O: 5 |
|  | 4569 | NX14046 | Rat | 1A | O: 5 |
|  | 4569 | NX14056 | Rat | 1A | O: 5 |
| 643 | 4580 | NX15096 | Sheep | 1A | O: 8 |
|  | 4580 | NX15098 | Sheep | 1A | O: 8 |
|  | 4581 | NX15097 | Sheep | 1A | O: 8 |
|  | 4581 | NX15100 | Sheep | 1A | O: 8 |
| 216 | 4596 | NX15132 | Pig | 1A | O: 5 |
|  | 4596 | NX17064 | Pig | 1A | O: 5 |
|  | 4596 | NX17067 | Pig | 1A | O: 5 |
| 541 | 4548 | NX12012 | Chicken | 1A | O: 5 |
|  | 4548 | NX12013 | Chicken | 1A | O: 5 |
| ST | CT | Samplename | Origin | Biotype | Serotype |
| 541 | 4756 | NX18106 | Human | 1A | O: 1,2,5 |
| 631 | 4739 | NX09043 | Pig | 1A | O: 8 |
|  | 4739 | NX09044 | Pig | 1A | O: 8 |
|  | 4739 | NX09045 | Pig | 1A | O: 8 |
| 145 | 4515 | NX09028 | Pig | 4 | O: 9 |
|  | 4515 | NX09029 | Pig | 4 | O: 9 |
| 157 | 4751 | NX12018 | Food | 1A | O: 9 |
|  | 4606 | NX18107 | Human | 1A | O: 9 |
| 162 | 4521 | NX09040 | Pig | 1A | O: 5 |
|  | 4747 | NX11036 | Pig | 1A | O: 5 |
| 166 | 4738 | NX09038 | Pig | 1A | O: 5 |
|  | 4595 | NX17063 | Pig | 1A | O: 5 |
| 389 | 4622 | NX19042 | Food | 2 | O: 9 |
|  | 4622 | NX19043 | Food | 2 | O: 9 |
| 563 | 4600 | NX18077 | Food | 1A | O: 53 |
|  | 4760 | NX19045 | Food | 1A | O: 9 |
| 600 | 4733 | NX09013 | Pig | 1A | O: 5 |
|  | 4745 | NX09058 | Pig | 1A | O: 9 |
| 625 | 4504 | NX0755 | Pig | 4 | O: 3 |
|  | 4532 | NX11058 | Pig | 4 | O: 3 |
| 633 | 4530 | NX09059 | Pig | 1A | O: 9 |
|  | 4742 | NX09061 | Pig | 1A | O: 9 |
| 634 | 4741 | NX09060 | Pig | 1A | O: 9 |
|  | 4582 | NX15099 | Sheep | 1A | O:5,8,9 |
| 635 | 4749 | NX12009 | Cattle | 1A | O: 5 |
|  | 4556 | NX12023 | Cattle | 1A | O: 5 |
| 636 | 4546 | NX12010 | Cattle | 1A | O: 5 |
|  | 4546 | NX12011 | Cattle | 1A | O: 5 |
| 642 | 4572 | NX14114 | Pig | 1A | O: 5 |
|  | 4578 | NX14121 | Pig | 1A | O: 5 |
| 5 | 4564 | NX12065 | Food | 1A | O: 8 |
| 6 | 4511 | NX09014 | Pig | 1A | O: 5 |
| 8 | 4762 | NX19048 | Food | 1A | O: 5 |
| 19 | 4734 | NX09017 | Pig | 1A | O: 5 |
| 219 | 4523 | NX09042 | Pig | 1A | O: 5 |
| 293 | 4557 | NX12024 | Cattle | 1A | O: 5 |
| 306 | 4601 | NX18080 | Food | 1A | Nag |
| 311 | 4554 | NX12019 | Food | 1A | O: 9 |
| 339 | 4566 | NX13077 | Pig | 1A | O: 9 |
| 360 | 4558 | NX12036 | Pig | 1A | O: 9 |
| 404 | 4735 | NX09018 | Pig | 1A | O: 5 |
| 415 | 4737 | NX09039 | Pig | 1A | O: 5 |
| 536 | 4588 | NX16044 | Cattle | 1A | O: 5 |
| ST | CT | Samplename | Origin | Biotype | Serotype |
| 626 | 4507 | NX09007 | Pig | 1A | O: 5 |
| 627 | 4508 | NX09008 | Pig | 1A | O: 5 |
| 628 | 4517 | NX09036 | Pig | 1A | O: 5 |
| 629 | 4736 | NX09037 | Pig | 1A | O: 5 |
| 630 | 4740 | NX09041 | Pig | 1A | O: 5 |
| 632 | 4748 | NX09048 | Pig | 1A | O: 5 |
| 638 | 4563 | NX12064 | Food | 1A | O: 8 |
| 639 | 4565 | NX13049 | Food | 1A | O: 5 |
| 641 | 4753 | NX14113 | Pig | 1A | O: 5 |
| 644 | 4619 | NX19005 | Food | 1A | Nag |
| 645 | 4628 | NX19106 | Human | 1A | Nag |
| 646 | 4597 | NX19112 | Food | 1A | O: 5 |
| 655 | 4743 | NX09057 | Pig | 1A | O: 8 |
| 656 | 4761 | NX19107 | Human | 1A | O: 8 |
| 26 | 4625 | NX19053 | Food | 2 | O: 9 |
| 18 | 4620 | NX19019 | Human | 4 | Nag |
